# Amish (Rural) vs. non-Amish (Urban) Infant Fecal Microbiotas Are Highly Diverse and Their Transplantation Lead to Differences in Mucosal Immune Maturation in a Humanized Germfree Piglet Model

**DOI:** 10.3389/fimmu.2019.01509

**Published:** 2019-07-16

**Authors:** Santosh Dhakal, Lingling Wang, Linto Antony, Jennifer Rank, Pauline Bernardo, Shristi Ghimire, Kathy Bondra, Christina Siems, Yashavanth Shaan Lakshmanappa, Sankar Renu, Bradley Hogshead, Steven Krakowka, Mike Kauffman, Joy Scaria, Jeffrey T. LeJeune, Zhongtang Yu, Gourapura J. Renukaradhya

**Affiliations:** ^1^Food Animal Health Research Program, Ohio Agricultural Research and Development Center, Wooster, OH, United States; ^2^Department of Veterinary Preventive Medicine, College of Veterinary Medicine, The Ohio State University, Columbus, OH, United States; ^3^Department of Animal Sciences, Ohio Agricultural Research and Development Center, The Ohio State University, Columbus, OH, United States; ^4^Department of Veterinary and Biomedical Sciences, South Dakota State University, Brookings, SD, United States; ^5^The Department of Veterinary Biosciences, College of Veterinary Medicine, The Ohio State University, Columbus, OH, United States

**Keywords:** gut microbiome, Amish and non-Amish infants, rural and urban microbiota, mucosal immune maturation, germfree pigs

## Abstract

The gut microbiome plays an important role in the immune system development, maintenance of normal health status, and in disease progression. In this study, we comparatively examined the fecal microbiomes of Amish (rural) and non-Amish (urban) infants and investigated how they could affect the mucosal immune maturation in germ-free piglets that were inoculated with the two types of infant fecal microbiota (IFM). Differences in microbiome diversity and structure were noted between the two types of fecal microbiotas. The fecal microbiota of the non-Amish (urban) infants had a greater relative abundance of Actinobacteria and Bacteroidetes phyla, while that of the Amish (rural) counterparts was dominated by Firmicutes. Amish infants had greater species richness compared with the non-Amish infants' microbiota. The fecal microbiotas of the Amish and the non-Amish infants were successfully transplanted into germ-free piglets, and the diversity and structure of the microbiota in the transplanted piglets remained similar at phylum level but not at the genus level. Principal coordinates analysis (PCoA) based on Weighted-UniFrac distance revealed distinct microbiota structure in the intestines of the transplanted piglets. Shotgun metagenomic analysis also revealed clear differences in functional diversity of fecal microbiome between Amish and non-Amish donors as well as microbiota transplanted piglets. Specific functional features were enriched in either of the microbiota transplanted piglet groups directly corresponding to the predominance of certain bacterial populations in their gut environment. Some of the colonized bacterial genera were correlated with the frequency of important lymphoid and myeloid immune cells in the ileal submucosa and mesenteric lymph nodes (MLN), both important for mucosal immune maturation. Overall, this study demonstrated that transplantation of diverse IFM into germ-free piglets largely recapitulates the differences in gut microbiota structure between rural (Amish) and urban (non-Amish) infants. Thus, fecal microbiota transplantation to germ-free piglets could be a useful large animal model system for elucidating the impact of gut microbiota on the mucosal immune system development. Future studies can focus on determining the additional advantages of the pig model over the rodent model.

## Introduction

The human gastro-intestinal (GI) tract is colonized with trillions of diverse microbes, which play crucial roles in host health and disease (1). Although being considered commensal, gut microbes are critical for the proper development of GI mucosa, mucosal immune system, and systemic immunity (1, 2). The diversity in GI microbiota is attributed to a host of factors including the environment and the interactions with host innate immune cells. Gut microbes and their fermentation products as well as other metabolites directly or indirectly influence GI and extra-intestinal (including respiratory) health and immunity (3–5). Dysbiosis of gut microbiota is associated with digestive tract disorders such as inflammatory bowel disease (IBD) and various immune, metabolic, and neuronal disorders including asthma, obesity, and autism (1, 6–9). Despite the similar genetic ancestries and lifestyles of Amish and Hutterite, the latter's children have a prevalence of asthma and allergic sensitization 4–6 times higher than Amish children (10). In contrast to Amish, the Hutterite follows industrialized farming practices, and the vast differences in the levels of allergens, microbiota, and endotoxins in indoor dust, in particular, were believed to attribute to the profound variations in the proportions, phenotypes, and functions of innate immune cells (10). The dust from the Amish homes, having different bacterial composition than that of the Hutterite homes, resulted in significantly lower airway allergic response in mouse model (10). Exposure to the diversity of the external microbial world ensures that many maladapted immune pathways leading to allergy, most if not all, can be counterbalanced (11).

Although the mouse model has provided crucial insights into the mechanism(s) regulating the immune systems that are mediated by the gut microbiota (12, 13), it remains to be determined how applicable the microbiota-induced immune response in germfree mice is to humans. Chung et al. compared small intestinal immune maturation in germfree mice colonized with either mouse or human fecal microbiota (HFM) and found that the latter led to very low levels of CD4^+^ and CD8^+^ T cells, proliferating T cells, dendritic cells, and secretion of antimicrobial peptides, all characteristics of germfree mice (14). Inoculation of HFM into germfree mice favored colonization of several strains of donor bacteria, but the important strains of the genera *Bifidobacterium, Lactobacillus*, and *Clostridium* present in the donor HFM did not persist in recipient mice (15–17). Other studies using mice could not precisely replicate the actual human-microbe relationship because mice are physiologically and metabolically distinct from humans (12, 13, 18). Mice do not display the clinical manifestations of the human enteric diseases, and some important human gut bacterial taxa fail to colonize in humanized mice (18). These limitations hinder research on human microbiome using mice for translational studies, including research on the relationship between the human microbiome and immune system development. Thus, to understand the role of certain important gut bacterial species and the effect of diverse commensal GI bacterial species on health and disease, a suitable large animal model that supports the growth and colonization of most of the important human gut bacteria is needed.

Swine are considered a suitable non-primate animal model for human microbiota-related studies (19), because pigs are anatomically, physiologically, and genetically more closely related to humans when compared to rodents (19–21). Immunologically, the distribution of lymphocytes at mucosal and systemic sites follows a similar pattern in humans and pigs; both have functional Peyer's patches where M cells perform antigen sampling function (18, 22). Pigs also have high similarities to humans in genomic and protein sequences, brain growth and development, and disease progression patterns (19). Moreover, the pig is a useful animal model system for various infectious diseases and the role of gut microbiota in modulating these infections (20). Piglets can be used for human gut microbiota studies and are ideal for identifying microbiome associated roles in infectious diseases such as rotavirus infection and immunity (23, 24). Thus, gut microbiota-transplanted piglets represent an alternative model to investigate human gut microbiota.

In this study, we delineated the influence of environmental conditions on the microbial diversity in the gut of children and mucosal immune development using a piglet model. We hypothesized that there would be vast differences in the microbial composition, structure, and functional diversity between Amish and non-Amish children, and the infant fecal microbiota (IFM) samples from each children group could be recapitulated by transplantation into a neonatal germ-free (Gf) piglet model and this would have a differential impact on mucosal immune development. The objectives of this study were to test the above hypotheses by comparing the IFM diversity of Amish (considered as rural) and non-Amish (considered as urban) children and to evaluate IFM colonization and the subsequent influence on immune maturation in a Gf piglet model.

## Materials and Methods

### Fecal Microbiota Sample Collection

Parents of the enrolled infants were informed of the nature and objectives of this study, and written consents were received in accordance with the sampling protocol approved by The Ohio State University Institutional Review Board (IRB). We enrolled five each of apparently healthy infants from Amish and non-Amish families. All the Amish households had farm animals (cattle, sheep, and/or horses) and pets (dog and/or cat), while the non-Amish households were located within the Wooster city limits and had no known contact with livestock but had a pet dog or cat (Table 1). One study has shown that the mode of delivery has a great impact on gut microbiota, with vaginal delivery resulting in greater diversity and species richness of gut microbiota than Cesarean section (25). For this reason, only infants who were born through natural vaginal delivery were enrolled in this study.

**Table 1:** Summary of the Amish and non-Amish children and their living house environment.

**Type of child**	**Age (months)**	**Animals raised by the family**	Breast feeding (months)^*^
Amish 1	14	Cow, horse, dog, cat	Breast (12)
Amish 2	15	Horse, dog, cat	Breast (13)
Amish 3	9	Cow, horse	Breast (3)
Amish 4	12	Cow, horse, sheep, chicken	Breast (10)
Amish 5	10	Horse, goat, chicken, dog, cat	Breast (9)
Non-Amish 1	10	None	Formula^**^
Non-Amish 2	9	None	Breast
Non-Amish 3	12	Dog	Formula^**^
Non-Amish 4	12	Dog	Not available
Non-Amish 5	11	Dog	Breast (11)

* Numbers in the parentheses indicate total months for which infants were either breast or formula fed.

** *These infants were not breast fed and were on formula from the time of birth*.

All IFM samples were collected from fresh soiled diapers as described previously (26, 27). Briefly, one teaspoon of fresh stool was transferred into a sterile 25-ml pre-weighted sterile screwcap bottle, which contained 5 g sterile glass beads (for homogenization), as soon as defecation was noted. Each of the bottles was then immediately filled completely with a sterile anaerobic medium to maintain anaerobiosis (23). Sealed bottles were placed on ice and transported to the laboratory within 30 min. The IFM samples were homogenized by vigorous vortexing. Following the addition of sterile glycerol to a final concentration of 15% (v/v), one ml aliquots were prepared in sealed cryopreservation tubes and immediately stored at −80°C.

### Fecal Microbiota Transplantation Into Gf Piglets, Husbandry, and Sample Collections

A healthy pregnant (gestation day−105) sow from The Ohio State University swine herd was procured and maintained in our isolation facility for 1 week prior to farrowing. Experimental Gf piglets were delivered by Cesarean-section as described in a previous publication (28). Piglets were maintained in individual temperature-controlled sterile germfree isolators and fed with a sterilized infant milk formula (Parmalat). Piglets were randomly grouped (male and female balanced) into two groups (*n* = 4 per group). Immediately before inoculation into piglets, two microbial inocula were prepared separately, with one being a mixture of the five Amish (rural) infant fecal microbiota (RIFM) aliquots (1.0 ml each) and the other being a mixture of the five non-Amish (urban) infant fecal microbiota (UIFM) aliquots (Table 1). Each inoculum was mixed with 40 ml sterile infant milk formula and orally administered to individual piglets of each group (referred to as RIFM-inoculated or UIFM-inoculated piglets, RIFMP, and UIFMP, respectively) at 2 weeks of age. Fecal inoculation was repeated twice at weeks 3 and 4 using similarly prepared inocula. Piglets were euthanized 3 weeks after the third inoculation (at 7 weeks of age, 5 weeks after the first fecal inoculation). Piglets were fed with ultrahigh temperature pasteurized formula milk (Parmalat brand), at increasing volume to meet the increasing nutritional requirements of the growing piglets as follows: 180 ml twice a day from days 0 to 5, 240 ml twice a day from days 6 to 20, 240 ml in the morning and 360 ml in the afternoon from days 21 to 30, and 240 ml in the morning and 480 ml in the afternoon from days 31 to 48. Fecal swab and blood samples were collected prior to the first inoculation, 4 days after the first inoculation, and on the day of necropsy. The fecal swab samples were collected and stored frozen (−80°C) until DNA extraction. Piglets were examined three times daily for any clinical sign of illness throughout the experiment. All the surgical procedures on the sow and experimental methods on the piglets were performed as per the approved protocols of the Institutional Animal Care and Use Committee at The Ohio State University.

At necropsy (pigs aged 49 days old), lumen digesta samples of ileum and colon were collected by extrusion. The intestines were then cut open longitudinally, and the mucosal surface was gently rinsed with sterile PBS to remove visible digesta contents. The intestinal mucosa was scraped off using sterile glass slides, collected into sterile tubes and stored at −80°C until DNA extraction. Mesenteric lymph nodes (MLN) and ileum tissues were collected in the DMEM medium containing antibiotics for isolation of mononuclear cells (MNCs) as described previously (28).

### Isolation of Mononuclear Cells (MNCs) and Flow Cytometry

Mononuclear cells (MNCs) of ileum and MLN were analyzed for immune cell frequencies by flow cytometry as described previously (29, 30). Briefly, cells were stained for cell membrane expressed markers T-helper cells (CD3^+^CD4α^+^), CD8 T cells (CD3^+^CD8α^+^), γδ T cells (CD3^+^ δ chain^+^), monocyte/macrophages (CD172^high^CD4α^−^), conventional dendritic cells (cDC, CD172^low^CD4α^−^), and plasmacytoid DC (pDC, CD172^low^CD4α^+^). Immunostained cells were evaluated and quantitated using the flow cytometer BD Aria II (BD Biosciences, CA) and the FlowJo software (Tree Star, OR) (29). Antibodies used were anti-porcine CD3 (Southern biotech, AL), CD4α (Southern biotech, AL), CD8α (Southern biotech, AL), δ chain (BD Pharmingen, CA), monocyte/granulocyte (CD172, Southern biotech, AL), anti-mouse CD79b (Bio-Rad, CA), anti-pig IgA (Bio-Rad, CA), anti-pig CD25 (Bio-Rad, CA) and anti-mouse FOXP3 (eBiosciences, CA). Respective isotype control monoclonal antibodies were included in the assay.

### Metagenomic DNA Extraction, 16S rRNA Gene Sequencing, and Sequence Analysis

Metagenomic DNA was extracted from the infant donor fecal samples and the piglet samples (fecal swabs, digesta, and mucosa of ileum and colon) using the repeated bead beating plus column purification (RBB+C) method as described previously (31). DNA integrity was examined by agarose (0.8%) gel electrophoresis, and DNA concentrations were determined using the Quant-iT^TM^ dsDNA Assay kit (ThermoFisher Scientific, Waltham, MA). The bacterial microbiota in all the fecal samples and the mucosal samples were characterized with respect to diversity, composition, and structure using high throughput sequencing of amplicon libraries of 16S rRNA genes and sequence analysis as described previously (32). Briefly, one amplicon library was prepared for each sample by PCR amplification of the V4 region using primers 515F and 806R, with each amplicon library having a unique dual index barcodes for multiplexing. After purification, the purified amplicon libraries of all the DNA samples were pooled at equal molar ratio and sequenced using the 2 × 300 paired-end chemistry on a MiSeq system. The raw sequencing data were deposited in SRA of GenBank with the accession numbers SRX4512314-SRX4512371.

The sequencing data were analyzed using QIIME 1.9.1 (http://qiime.org/) as described previously (33). Briefly, the bases with a quality score < 25 were trimmed off from each sequencing read, and then the quality-checked sequences shorter than 248 bp after trimming the barcodes and primers were discarded. The paired reads were joined to form a single sequence using fastq-join (34). Probable chimeric sequences were identified using ChimeraSlayer (35). Species-equivalent operational taxonomic units (OTUs) were clustered by comparing the sequences to the Silva_119_release reference sequences (http://www.arb-silva.de/download/archive/qiime/) at 97% similarity using the UCLUST algorithm (36). Each OTU was taxonomically classified according to the same reference database. Minor OTUs were filtered out if they were each represented by <0.005 % of the total sequences (37) or <0.1% of the sequences in any of the individual samples.

### Microbial DNA Enrichment, Shotgun Metagenome Sequencing, and Analysis

The metagenomic DNA from the infant donor fecal samples and the piglet colon content samples was processed further to selectively enrich the microbial DNA using the NEBNext® Microbiome DNA Enrichment Kit (New England Biolabs, Inc. MA) according to manufacturer's protocol (38). In brief, 40 μl of MBD2-Fc protein bound magnetic beads were used to separate eukaryotic DNA from 0.25 μg total metagenomic DNA of each sample. The magnetic beads and the attached eukaryotic DNA were removed with the help of a magnetic rack, and the supernatant containing the microbial DNA was then purified using Agencourt AMPure XP beads (Beckman Coulter). Sequencing libraries were prepared from the enriched and purified microbial DNA using a Nextera XT DNA Sample Prep Kit (Illumina Inc. San Diego, CA) per manufacturer's protocol. Bead-normalized libraries were pooled in equal amounts and sequencing was performed on a Miseq platform (Illumina Inc. San Diego, CA) using the 2 × 250 paired-end chemistry. The DNA samples from the piglet fecal swabs, mucosa of ileum, colon, and digesta of ileum were not subjected to metagenomic sequencing due to the lack of sufficient quantity. The shotgun metagenomic sequence data can be accessed at NCBI SRA under the bioproject “Rural and Urban infant's fecal microbiota study” (bioproject accession # PRJNA484151).

Taxonomic and functional profiling of the metagenomic samples were performed using MG-RAST (Metagenomic Rapid Annotations using Subsystems Technology) and the “representative hit” classification method (39). Briefly, the paired reads in FASTQ format were joined to form individual single sequences using the CLC genomics workbench (v11.0.0, Qiagen) before uploading to MG-RAST. Quality assessment, pre-processing of sequences to trim the low-quality regions from FASTQ data, de-replication to remove artificial duplicate reads (ADRs), and removal of host-specific sequences was performed in MG-RAST to ensure sequence quality. Taxonomic and functional profiles of the metagenomes were generated in MG-RAST using the RefSeq and the SEED subsystem databases, respectively. OTU tables were generated based on the representative hit classification method and an *e* value of ≤ 10^−5^. OTUs that passed the criteria of 60% identity over a minimum alignment length of 50 amino acids and were each represented by a minimum of 100 sequences were selected for building OTU relative abundance tables.

### Statistical Analysis

From the 16S rRNA gene sequence data, alpha diversity measurements were calculated using Qiime and compared between the two groups of infants and their, respectively, humanized piglets using unpaired *t*-test. The beta-diversity comparison of the microbiota between the different groups was examined using principal coordinates analysis (PCoA) based on weighted UniFrac distance matrix calculated from the 16S rRNA gene sequences, and ANOSIM (40) was used to access if significant difference (declared at *P* < 0.05) was detected. The relative abundance of individual taxa was compared between the two groups of infants and their, respectively, humanized piglets using linear discriminant analysis effect size (LEfSe) at (http://huttenhower.sph.harvard.edu/galaxy/). The normalization of sample abundance and LDA effect size calculation were carried out using the default parameter of Galaxy/Hutlab web pages: 0.05 for alpha value for the factorial Kruskal-Wallis test among classes and 2.0 for threshold on the logarithmic LDA score for discriminative features.

The relative abundance of immune cells was compared between the two groups using the unpaired *t*-test. Spearman correlation coefficients were calculated to assess correlations between individual taxa (genera and OTUs) and immune cells data were analyzed using SAS (SAS Institute Inc., Cary, NC) and visualized using the corrPlot package in R. Statistical significance was declared at *P* < 0.05, while statistical trend was considered at 0.051 < *P* < 0.10.

From the shotgun metagenomic sequence data, the relative abundance of individual OTUs and functional features at SEED subsystem level 3 was compared using linear discriminant analysis effect size (LEfSe). The beta-diversity comparison based on Bray-Curtis dissimilarity (both OTUs and functional features at subsystem level 3) was performed using PCoA. ANOSIM (40) was also used to access if significant difference (declared at *P* < 0.05) was detected between the groups. Two-sided White's non-parametric *t*-test combined with Story's false discovery rate (FDR) multiple test correction method was used to identify significant features at 95% confidence (41, 42).

## Results and Discussion

### Amish and non-Amish Infants had Substantial Differences in Their Fecal Microbiota

The average age of Amish and non-Amish infants included in this study was 12 and 10.8 months, respectively (Table 1). All of the Amish infants were breast-fed while at least two of five non-Amish infants were only formula-fed. The Amish families mainly differed from non-Amish families in terms of their involvement in agricultural practices and exposure to farming environment. Amish families raised and lived in close proximity to farm animals including cow, horse, sheep, and goat while non-Amish families either had dog(s) as pet animal or no animals in their households (Table 1). Amish families grew vegetables and agricultural produces for daily consumption while non-Amish households largely depended on purchased food supplies. The resulting differences in dietary habits are likely to account for the microbiota changes between these two groups but detailed diet information is not available and is out of the scope of this study.

**Figure 1:**
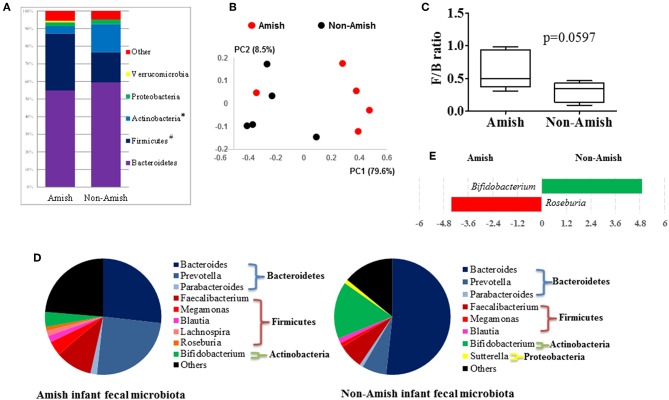
Comparison of the fecal microbiota between Amish and non-Amish infants. **(A)** Bacterial phyla identified, ^*^significantly lesser relative abundance in Amish than in non-Amish, ^#^significantly greater relative abundance in Amish than in non-Amish; **(B)** principal coordinates analysis (PCoA) plot; **(C)** Firmicutes/Bacteroidetes ratio; **(D)** major bacterial genera (each represented by >1% of total sequences); **(E)** LEfSe plot at the genus level.

On an average, nearly 34,000 quality-checked 16S rRNA gene sequences were obtained per sample, ranging from 18,696 to 49,258 sequences (Table S1). Of these sequences, 23,710 sequences/sample on an average (ranging from 11,693 to 41,150 sequences per sample) were assigned to major OTUs. The majority of the OTUs found from the donor fecal samples of both the Amish and non-Amish infants were assigned to the genera of Actinobacteria, Bacteroidetes, Firmicutes, and Proteobacteria, and together they accounted for an average of 94.4% of all the sequences (Figure 1A). The high predominance of these phyla is consistent with previous studies (43, 44). The non-Amish infants had a significantly higher relative abundance of Actinobacteria (15.9 vs. 4.15%) and higher, but statistically non-significant, relative abundance of Bacteroidetes (59.5 vs. 54.9%) than the Amish peers, while the latter had a significantly higher predominance of Firmicutes (32.3 vs. 17.1%). The greater abundance of Actinobacteria in non-Amish infants might also be contributed by probiotics-enriched formula since at least two of the five infants were formula-fed in non-Amish group. The two infant groups did not differ in the predominance of Proteobacteria (Figure 1A). The ratio of Firmicutes to Bacteroides (F/B ratio) tended (*P* = 0.060) to be higher among the Amish than among the non-Amish infants (0.62 ± 0.3% vs. 0.3 ± 0.16%, Figure 1C).

Large differences in the overall fecal microbiota were revealed by PCoA using Weighted-UniFrac distance matrix between the Amish and the non-Amish cohorts (Figure 1B); the differences were mainly attributed to differences in relative abundance of the phyla Actinobacteria, Firmicutes, and Bacteroidetes (Figure 1A). The Amish infants had a greater species richness than the non-Amish infants (Table 2). This is consistent with the finding of a previous study that revealed a greater diversity in the gut microbiota of adult Malawians, Amerindians, and Natives Americans who lived in rural areas than those who lived in urban areas (45). Differences in lifestyle and exposure to different sources of environmental microbes likely attributed to these differences.

**Table 2:** Alpha diversity measurements of fecal samples of rural and urban infants.

**Measurements**	**Amish**	**Non-Amish**
Number of OTUs observed	91^a^	68^b^
Chao1 estimate	98 ± 17^a^	72 ± 14^b^
Shannon diversity index	2.46 ± 0.34	2.58 ± 0.30
Simpson index of diversity	0.80 ± 0.08	0.85 ± 0.03
Evenness	0.54 ± 0.06	0.60 ± 0.06

*The values are presented by mean or mean ± sd. Different superscripts within a row indicate significant difference (P < 0.05), as assessed by unpaired t-test*.

### Amish and non-Amish Infant Fecal Microbiota Resembled the Rural and Urban-Type Infant Microbiota Described in Published Literature

In the Amish and non-Amish infant fecal samples, we identified 31 and 26 genera of bacteria, respectively, with 22 and 16 of them, respectively, having a relative abundance >0.1% (Tables S2a, S3a). A previous study, using Illumina high-throughput sequencing and biochemical analysis, revealed 17 and 15 genera with greater than 0.1% abundance respectively in breast-fed and formula-fed infant feces (43). *Parabacteroides, Prevotella, Faecalibacterium, Megamonas*, and *Roseburia* tended to be more predominant among the Amish infants than among the non-Amish infants, while *Bacteroides, Bifidobacterium*, and *Sutterella* showed the opposite trend (Figure 1D). Based on LEfSe analyses, however, only two genera were significantly different in relative abundance between the two groups of infants, with *Bifidobacterium* being enriched in non-Amish infants and *Roseburia* enriched in Amish infants (Figure 1E).

The genera *Prevotella, Bacteroides*, and *Parabacteroides* are closely related taxonomically within the phylum Bacteroidetes. A previous study comparing the fecal microbiota of African villager in Burkina Faso and European children showed exclusive detection of *Prevotella* in African villagers and a predominance of *Bacteroides* in the European infants (44). The predominance patterns of *Prevotella* and *Bacteroides* we observed among the Amish and non-Amish infants concur with the findings of the above study. Actually, similar trends were also observed in other studies comparing rural and urban children, and dietary and environmental factors were considered responsible for such differences (46, 47). People living in urbanized communities consume diets rich in animal fats and proteins, which *Bacteroides* spp. utilizes for their growth (46, 48). In contrast, rural communities consume diets rich in vegetables and grains, and *Prevotella* spp. is dominant in their gut to aid degradation of hemicellulose and other fibrous polysaccharides (47, 48). In our study, both Amish and non-Amish families lived in close proximity in Ohio, USA. However, Amish people are involved in farming and live in farm environment and differ from non-Amish urban communities in their socio-cultural practices (49). The differences between these two groups of subjects could be attributed to diet, exposure to the farm environment, and differences in socio-cultural practices. Thus, Amish infant gut microbiota resembles the “rural-type” microbiota, while the non-Amish urban infant gut microbiota represents “urban-type” microbiota. Previous reports have shown that Amish children exhibit a low incidence of asthma and allergies, similar to the children from rural “farm” communities in the developing world (10, 50, 51). Non-Amish urban children are highly prone to allergic and other adverse health events, which are attributed to modernization resulting in reduced and/or absence of most of the beneficial gut microbes and colonization from early stages of infancy (10, 51, 52). Collectively, our study demonstrated clear differences in the gut microbiota composition between Amish and non-Amish infants which might differentially influence mucosal immune system development and health issues such as asthma and food allergies, possibilities worthy of further research.

### Gf Piglets Transplanted With Amish/rural and non-Amish/urban Infant Fecal Microbiota Exhibit Differences in Their Gut Microbiota Profiles

**Figure 2:**
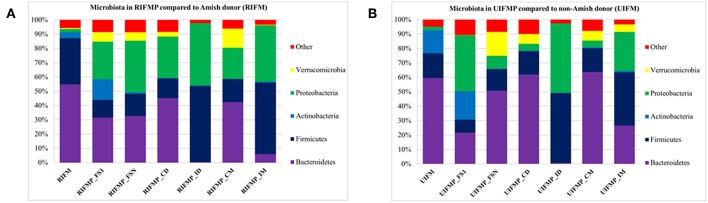
Bacterial phyla detected in the fecal microbiota of infants and the transplanted piglets. RIFM and UIFM, Amish and non-Amish infant fecal microbiota, respectively; RIFMP and UIFMP, piglets transplanted with RIFM and UIFM, respectively; FS1 and FSN, fecal samples collected at 4 days after the first inoculation and at necropsy, respectively; CD, colonic digesta; ID, ileal digesta; CM, colonic mucosa; IM, ileal mucosa. All the ileal and cecal samples were collected at necrospy. Both **(A)** and **(B)** were based on 16S amplicon sequences.

We compared the microbiome of RIFM- and UIFM-inoculated piglets to examine if the two types of transplanted IFM resulted in different gut microbiota in the humanized piglets. Four days after the first IFM inoculation, all the bacterial phyla found in the inocula were found in the fecal swabs from both groups of transplanted piglets although their relative abundance varied (Figures 2A,B). Because pigs are an outbred species, variations in bacterial colonization profiles among individual transplanted piglets were expected and were found (data not shown). In a previous study, a fecal suspension of a 10-year old boy was orally transplanted to neonatal Gf pigs, and a microbiota similar to the human inoculum was observed in the piglet 5 days post transplantation, which became stable by 12 days after transplantation (23). In another study, colonization by bacterial microbiome rich in *Proteobacteria* was detected in pigs within 24 h after transplantation with HFM (18). Also consistent with an earlier study (18), we found that *Proteobacteria* predominated in the Gf piglets shortly after transplantation (4 days).

**Figure 3:**
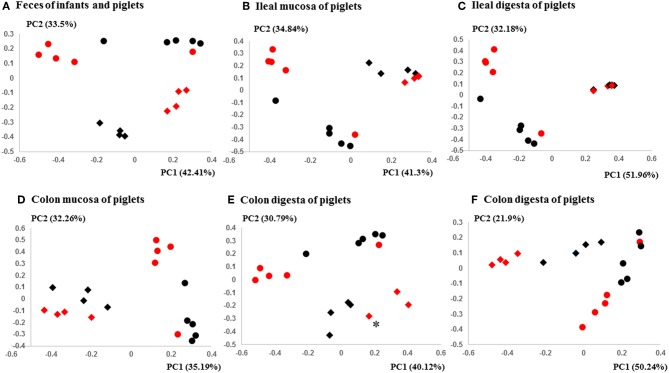
PCoA plots comparing the fecal microbiota of Amish and non-Amish infants and intestinal microbiota of transplanted piglets. **(A–E)** were based on 16S amplicon sequences and **(F)** was based on Bray-Curtis dissimilarity at genus level identified using shotgun metagenomic sequences. The infant fecal microbiota (IFM) was included in all of the plots to aid comparison (red spheres = rural IFM, black spheres = urban IFM). Microbiota of transplanted piglets in different samples (**A**, Feces; **B**, Ileal mucosa; **C**, Ileal digesta; **D**, Colon mucosa; **E**, Colon digesta; and **F**, Colon digesta) are represented by diamonds [red = rural IFM transplanted pig (RIFMP) and black = Urban IFM transplanted pig (UIFMP)]. The ileum and colon samples were collected at necropsy. The distance matrix between samples was calculated using Weighted-UniFrac and ANOSIM analysis revealed significant difference (*P* < 0.05) between the rural and the urban groups of IFM and the respectively humanized groups of piglets **(A–E)**. ANOSIM revealed significant difference (*P* < 0.05) in the fecal samples of piglets, but not in IFM between the rural and the urban groups based on shotgun metagenomic analysis **(F)**. ^*^Indicated that two plots were too close to distinguish.

The PCoA indicated statistically non-significant difference (*P* > 0.05) in the overall fecal microbiota composition or structure between the two groups of piglets after the initial inoculation (data not shown). However, 5 weeks after transplantation (the day of necropsy), significant differences (*P* < 0.05) were observed in the fecal, ileal (both mucosal and digesta), and colonic (both mucosal and digesta) samples as revealed by both 16S sequence analysis and shotgun metagenomics (Figure 3). Of the detected phyla, Bacteroidetes had a greater relative abundance in the IFM and the fecal and colonic samples of all the humanized piglets, but not in the ileal samples, which had Firmicutes as the largest phylum. Bacteroidetes was also more predominant in the samples of the UIFM-transplanted piglets (UIFMP) than the RIFM-transplanted piglets (RIFMP) (Figures 2A,B). Proteobacteria and Verrucomicrobia were more predominant, while Actinobacteria was less predominant, in the two groups of piglets than in the respective IFM inocula 5 weeks after the transplantation (Figures 2A,B), indicating that Proteobacteria and Verrucomicrobia were better adapted to the gut of the piglets than Actinobacteria.

Of the genera detected in the RIFM and UIFM inocula, 8 and 7 were, respectively, detected as major genera (Tables S2a, S3a). Many of these genera, including *Bacteroides* and *Bifidobacterium*, both of which also colonized successfully in Gf piglets in a previous study (23), were found in both the inocula and the humanized piglets. However, some genera were only found in the inocula, including two predominant genera, *Faecalibacterium* and *Prevotella*. On the other hand, some genera were detected in the piglets but not in the inocula. These genera might represent those that were more competitive in the gut of piglets. The above discrepancies between the donor inocula and the piglets have been reported in other studies (18, 25, 53), and they are probably attributable to the dietary and genetic differences, among other factors, between the infants and the piglets. Nevertheless, these results suggest that gut microbiota development can be primed or intervened using fecal microbiota inoculation.

**Figure 4:**
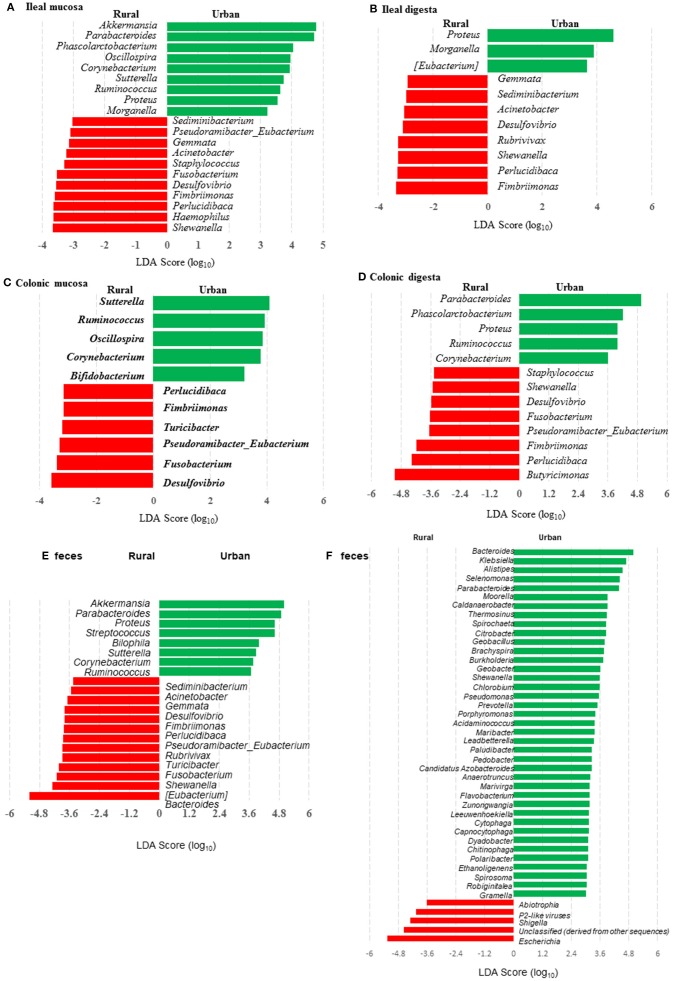
LEfSe plots showing the genera differing significantly in relative abundance between the two groups of infants and the humanized piglets. **(A-E)** were based on amplicon sequences of 16S rRNA genes, while **(F)** was based on shotgun sequences of colon digesta of the piglets.

Many genera were found to have opposite trends of occurrence in the fecal and the gut microbiota between the two groups of piglets (Figures 4A–F). Although not all the genera had consistent occurrence in the rural vs. urban groups, several of them were much more predominant in only one of the two groups. Specifically, in RIFMP but not UIFMP, *Acinetobacter* was substantially enriched in both ileal mucosa and digesta, while *Desulfovibrio, Fimbriimonas*, and *Perlucidibaca* were substantially enriched in all the samples, and *Fusobacterium, Puedorambacter-Eubacterium, Sedimenibacterium*, and *Shewanella* were substantially enriched in at least three of the five types of samples. In contrast, in UIFMP but not RIFMP, *Morganella* was considerably enriched in the ileal mucosa and digesta, while *Parabacteroides, Proteus, Ruminococcus*, and *Sutterella* were dramatically enriched in at least three of the five types of samples. Intriguingly, none of the above genera that were enriched in RIFMP was predominant in the corresponding inoculum (Table S2a); they were either very minor (*Desulfovibrio, Fusobacterium*) or undetected (*Acinetobacter, Fimbriimonas, Perlucidibaca, Pseudorambacter*-*Eubacterium, Sedimenibacterium*, and *Shewanella*) in the fecal microbiota of Amish infants. However, three (*Parabacteroides, Ruminococcus*, and *Sutterella*) of the five genera enriched in UIFMP were predominant in the corresponding inoculum although the remaining two genera were not detected in the fecal microbiota of non-Amish infants (Table S3a). When the genera detected in the metagenomic sequences of the colon digesta samples were compared between the two groups of piglets, more genera appeared enriched in the urban group than in the rural group (Figure 4F). Most of these differential genera were not detected by the 16S amplicon sequences (Figure 4D). Such discrepancy is commonly reported in the literature (54, 55). Nevertheless, given the difference in only two genera in the inocula (Figure 1E), the gut microbiota in the humanized piglets appeared to have expanded the difference in the fecal microbiota inocula, at least at the genus level. These results suggest that germ-free piglets are a useful large animal model in which differential human gut microbiota can be manifested.

More OTUs were detected in the ileum mucosa/digesta, colon mucosa/digesta and fecal samples of RIFMP than in the respective samples of UIFMP (125–168 vs. 85–141) (Tables S2b, S3b, Table 3). This mirrors the species richness (160 OTUs in Amish vs. 137 OTUs in non-Amish feces) in the corresponding inocula from the donors (Tables S2b, S3b and Table 4). These results suggest that the gut microbiota of humanized piglets can reflect that of the inocula at the OTU level. The majority of the OTUs that were found in the inocula were able to colonize the gut of the piglets. This includes some of the major OTUs assigned to *Bifidobacterium* and *Bacteroides*. However, as in the occurrence at the genus level, some of the OTUs in the inocula were not detected in the piglets, including some OTUs in the genera *Blautia, Faecalibacterium*, and *Prevotella*, while the opposite also holds true for other genera. Again, this is expected and can be attributed to the differences in gut environment and nutrients available between the infants and the piglets.

**Table 3:** Alpha diversity measurements in humanized pig fecal samples.

**Measurements**	**RIFMP**	**UIFMP**
Shannon diversity index (*H'*)	2.61 ± 0.45	2.61 ± 0.29
Simpson index of diversity (iD)	0.82 ± 0.08	0.82 ± 0.06
Chao1 estimate	115 ± 9^a^	90 ± 5^b^
Evenness	0.55 ± 0.10	0.55 ± 0.06

*RIFMP and UIFMP, piglets transplanted with fecal microbiota of rural and urban infants, respectively. The values are presented by mean ± sd. Different superscripts indicate significant difference (p < 0.05), as assessed by unpaired t-test*.

**Table 4:** The alpha diversity measurements of ileal and colonic microbiota of the humanized piglets.

	**Ileal mucosa**		**Ileal digesta**		**Colon mucosa**		**Colon digesta**	
	**RIFMP**	**UIFMP**	**RIFMP**	**UIFMP**	**RIFMP**	**UIFMP**	**RIFMP**	**UIFMP**
Shannon	2.04 ± 0.30	2.65 ± 0.50	1.49 ± 0.32	1.91 ± 0.37	2.50 ± 0.36	2.71 ± 0.15	0.48 ± 0.03	0.50 ± 0.05
Simpson	0.71 ± 0.11	0.84 ± 0.11	0.62 ± 0.09	0.76 ± 0.11	0.84 ± 0.05	0.83 ± 0.03	0.87 ± 0.02	0.86 ± 0.02
Chao1	130 ± 12	117 ± 6	81 ± 17	57 ± 16	114 ± 15	104 ± 4	117 ± 18^a^	88 ± 9^b^
Evenness	0.42 ± 0.06	0.56 ± 0.10	0.34 ± 0.06^a^	0.47 ± 0.08^b^	0.53 ± 0.07	0.58 ± 0.03	0.10 ± 0.01	0.11 ± 0.01

*RIFMP and UIFMP, piglets transplanted with fecal microbiota of rural and urban infants, respectively. The values are presented by mean ± sd. Different superscripts indicate significant difference (P < 0.05) between rural and urban samples as assessed by unpaired t-test*.

When the occurrence of the major OTUs was compared between the two groups of piglets using LEfSe, 145 OTUs were found to be differential in at least one sample between the two piglet groups (Table S4). These OTUs are taxonomically diverse, but UIFMP has more enriched OTUs assigned to *Parabacteroides* and *Bacteroides* than RIFMP, whereas RIFMP had more enriched OTUs distributed in many taxa, including *Myxococcales, Clostridiales, Fimbriimonas*, candidate family S24-7, *Ruminococcaceae, Lachnospiraceae*, and *Bradyrhizobiaceae*. Only five OTUs were differential between the two groups of infants, and the two OTUs enriched in UIFM were also enriched in UIFMP. However, the three OTUs enriched in RIFM were not particularly enriched in RIFMP. Again, the microbiota differences in the inocula became more pronounced inside the gut of the piglets. Future research is needed to determine the ability of different bacteria to colonize the gut of Gf piglets and how differences in the fecal inocula affect the gut microbiota in Gf piglets after inoculation.

### Bacterial Colonization Varied Substantially in Different Segments of the Intestines of Piglets After Inoculation

The relative abundance of individual bacterial taxa in the transplanted piglets varied substantially between the ileum and the colon (Figure 2). For example, Firmicutes was more predominant in the ileum of the transplanted piglets, while Bacteroidetes predominated in the colon. This result is consistent with previous studies (56, 57). Fecal microbiota better represents the colonic microbiota than the ileal microbiota (7, 56). Similarly, we also observed that the relative abundance of different phyla in the fecal samples of the transplanted piglets was comparable to that in the colon but not the ileum, regardless of the source of the donor inoculum (Figure 2). Moreover, differences among the different gut segments and feces were also evident at both the genus (Tables S2a, S3a) and OTU (Tables S2b, S3b) levels. Although microbial diversity along the GI tract followed similar trends in both the RIFMP and UIFMP, there were dramatic differences in terms of colonization of different OTUs and genera at different GI sites (Figure 4, and Table S4). Future research is needed to determine how the difference in fecal microbiota can be manifested in different segments of the gut in Gf piglets after inoculation.

### Metagenomic Analysis Showed Clear Differences in Taxonomic and Functional Diversity of Fecal Microbiome Between the Two Groups of Infants and Respectively Humanized Piglets

We compared the microbiomes of both the donor inocula and the colon digesta of the piglets using shotgun metagenomic sequencing to examine (i) how the differences in the microbiome between the two infant donor groups were manifested in the piglet recipients and (ii) how the shotgun sequencing data complemented the 16S rRNA gene-based analysis. PCoA based on Bray-Curtis dissimilarity reiterates the distinct bacterial microbiome differences between the two groups of piglets at the genus level (Figure 3F). Proteobacteria and Bacteroidetes dominated in the colon digesta of the transplanted piglets whereas Bacteroidetes and Firmicutes dominated in the donor inocula (data not shown). Consistent with the results of the 16S rRNA gene-based analysis, Proteobacteria was significantly (*q* ≤ 0.05) more predominant in the colon digesta of RIFMP, while Bacteroidetes was significantly (*q* ≤ 0.05) more predominant in the colon digesta of UIFMP. A comparison using LEfSe showed clear difference in many genera of bacteria in the colon digesta between the two groups of piglets, with more genera (including *Bacteroides, Parabacteroides, Klebsiella, Alistipes*, and *Prevotella*) enriched in the colon digesta of UIFMP (Figure 4F). The genus *Escherichia* showed a significantly greater relative abundance in the colon digesta of RIFMP than in the UIFMP. It is intriguing that the inoculation with the urban infant fecal microbiota resulted in enrichment of that many genera of bacteria in the colon of the piglets.

**Figure 5:**
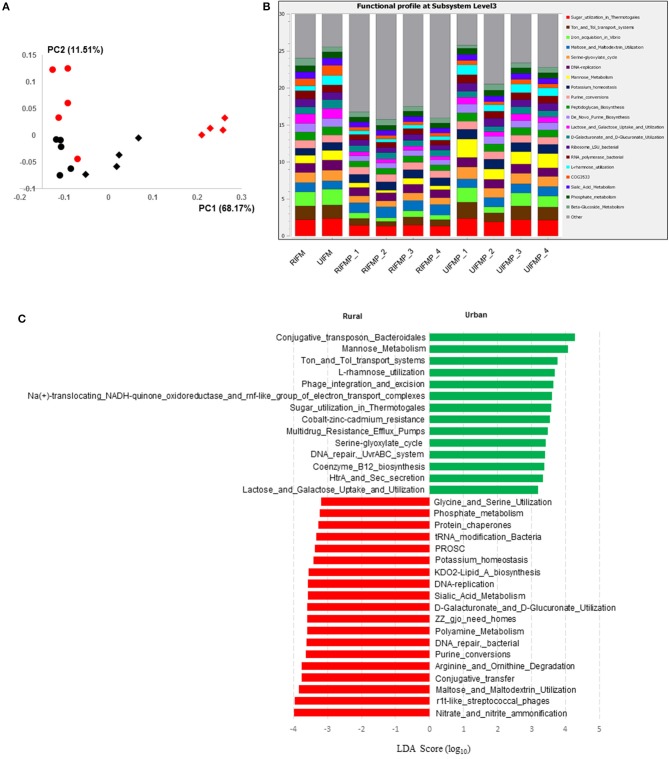
Functional profiling of colon digesta microbiota of piglets humanized with rural and urban infant fecal microbiota. **(A)** PCoAplot (using Bray-Curtis dissimilarity). Spheres, infant fecal microbiota (red, rural; black, urban). Diamonds, colonic samples of piglets (red, RIFMP; black, UIFMP). ANOSIM indicated significant difference between rural and urban groups (*p* < 0.05) of both the infant fecal microbiota and feces of, respectively, humanized piglets. **(B)** Stacked bar plot illustrating the 20 most abundant functional features (at subsystem level 3) represented by different colors. **(C)** A LEfSe plot showing the significantly different *(p* < 0.05) functional features (at subsystem level 3) between the two piglet groups. Only the top 50 major function were analyzed. RIFM and UIFM represent Amish/rural-type IFM and non-Amish/urban-type IFM, while RIFMP and UIFMP represent RIFM- and UIFM-transplanted piglets, respectively.

The functional capacity of the metagenome of both the infant donor inocula and the piglet colon digesta was assessed by annotating the protein-coding genes predicted from the metagenomic sequences and searching against SEED subsystem database using MGRAST. Overall, the colon digesta metagenome of the two groups of piglets had different functional diversity (Figure 5A). The 20 most predominant functional features are shown in Figure 5B. Of the major functional features (each represented by ≥100 sequences), 477 were significantly different (*q* ≤ 0.05) between the two piglet groups, with 370 differing with a *q* ≤ 0.01. These differential functional features were further analyzed using LEfSe to identify the enriched functions in each piglet group (Figure 5C). The functions enriched in the colon digesta microbiome of UIFMP, when compared to that of RIFMP, included conjugative transposon and phage infection, sugar (mannose, L-rhamnose, lactose, and galactose) utilization, Ton and Tol transport systems, metal and drug resistance, electron transport complex, DNA repair, B12 synthesis, and secretion. The TonB-dependent receptor, β-galactosidase (E.C 3.2.1.23) and integrase are protein families found significantly more abundant in the UIFMP than in the RIFMP. The relative abundance of the TonB-dependent receptor in the urban UIFMP might be an indication of successful colonization of *Bacteroides*, whose genome is enriched with these receptors (58, 59). On the other hand, the colon digesta microbiome of RIFMP was enriched for a variety of functions including nitrate/nitrite reduction, phage, maltose, and maltodextrin metabolism, and amino acid metabolism, phosphate metabolism, protein folding, and amino acid metabolism. Maltodextrin is a class of glucose polymers preferred by Proteobacteria in both mammalian hosts and in the environment. The maltose/maltodextrin transport system, a member of ABC high-affinity transport systems of enteric bacteria, is responsible for the uptake of these sugars, and the utilization of these substrates is well-studied (60, 61). The predominance of this functional feature in the RIFMP reflects the expansion of Proteobacteria in those piglets. The differences in functional features are consistent with the taxonomic differences, but future research is needed to explore how such functional differences affect the development of the gut and other organs of the host.

### Some Bacteria That Colonized the Transplanted Piglets Correlated to Modulations in Important Immune Cell Subsets in Mucosal Tissues of the Transplanted Piglets

**Figure 6:**
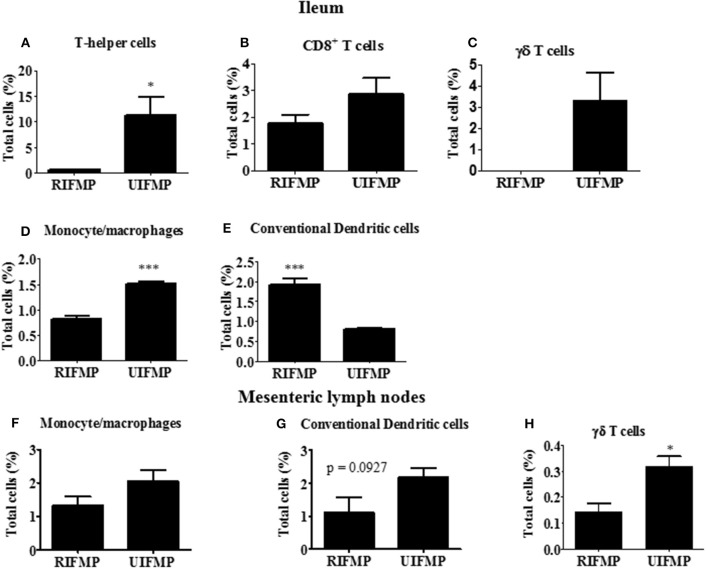
Various immune cells in the mucosa of humanized piglets. **(A)** T helper cells (CD3^+^CD4α^+^}, **(B)** CD8^+^ T cells (CD3^+^CD8α^+^), **(C)** γδ T cells, **(D)** Monocyte/Macrophages, and **(E)** conventional dendritic cells (cDC) (CD172^low^CD4α^−^) in ileum; and **(F)** Monocyte/Macrophages; **(G)** cDC, and **(H)** γδ T cells in MLN. Data are presented as the mean ± SEM of 4 piglets analyzed using unpaired *t*-test. Asterisks denote significant difference (^*^P < 0.05, and ^***^P < 0.001). A similar trend in immune cell frequencies in the ileum and MLN were observed in another similar independent experiment in Gf piglets (data not shown). RIFMP and UIFMP represent rural-type IFM-transplanted piglets and urban-type IFM-transplanted piglets, respectively.

We determined the relative abundance of various lymphoid and myeloid immune cells in the ileum and MLN of piglets (Figure 6). The RIFMP had significantly lower (*P* < 0.05 frequencies of T helper cells (CD4^+^ T cells) and monocytes/macrophages, but higher (*P* < 0.001) frequency of conventional dendritic cells (cDC) in the ileum compared to the UIFMP (Figures 6A,D,E). The frequency of CD8^+^ T cells and γδ T cells tended to be lower in the RIFMP (Figures 6B,C). In MLN derived MNCs, cDC tended to be higher (*P* = 0.0927) among the UIFMP than the RIFMP (Figure 6G). The frequency of monocytes/macrophages was in higher trend and γδ T cells were significantly increased (*P* < 0.05) in the UIFMP (Figures 6F,H). Gf animals are typically Th2–skewed, and metabolites produced by different bacteria can affect the maturation and function of DCs (62). The reduced frequency of lymphoid cells and macrophages and the increased cDCs in the small intestine of the RIFMP compared to UIFMP indicate robust activation of cDCs in the gut of pigs mediated by RIMP. cDC is the principal subset of DCs inducing Th2 cell-mediated immunity in the lymph nodes involved in orchestrating allergic inflammation in the lung (63). cDC is essential for the induction of primary T and B cell responses in the gut mucosa, specifically Th2-associated responses (64). Depending on the type of macrophages activated (M1 or M2 type) they play an important anti-inflammatory role and can decrease immune reactions through the release of cytokines (65). This indicates that RIFMP (but not UIFMP) mediated mucosal immune development likely enhance resistance against pathogens and maintain immune homeostasis/tolerance against allergic conditions. However, due to the lack of direct evidence for such early immune development mediated through diverse intestinal microbiome and the consequence of health and disease, future investigations should be directed toward confirming the benefits of differential immune activation by diverse gut microbiome against microbial infections and allergic reactions in the infant pig model.

**Figure 7:**
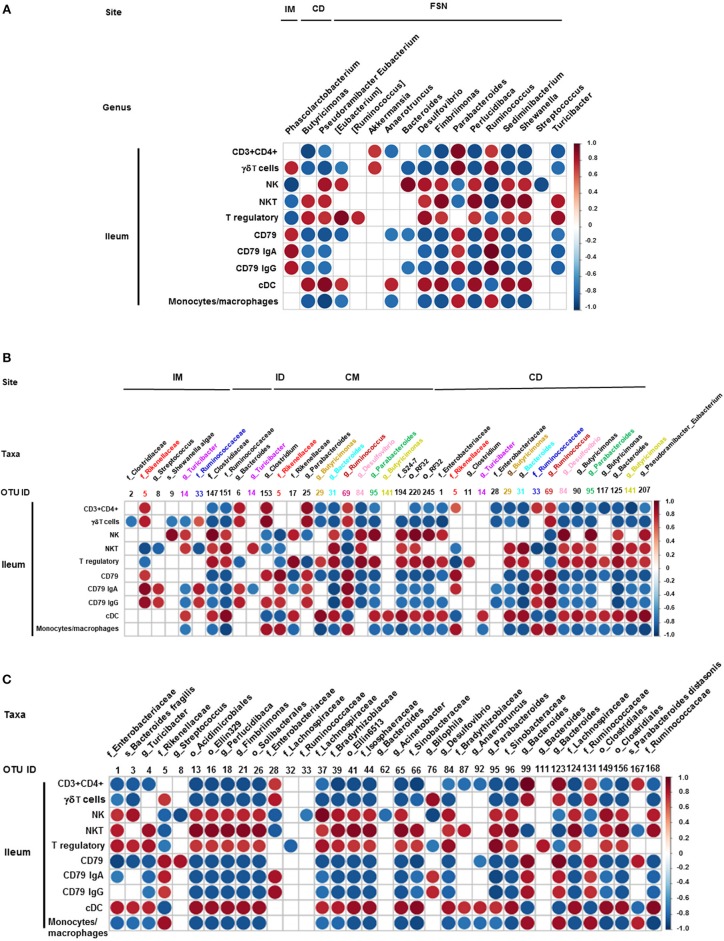
Correlation between ileal immune cells and individual bacterial taxa of the transplanted piglets. **(A)** Correlation of different immune cells of ileum with bacterial genera in ileum mucosa (IM), colonic digesta (ID), and feces collected at necropsy (FSN). **(B)** Correlation between different ileal immune cells with bacterial OTUs present at ileum mucosa (IM), ileum digesta (ID), colonic mucosa (CM), and colonic digesta (CD). **(C)** Correlation between different ileal immune cells with bacterial OTUs in the fecal samples collected at necropsy. The color of the circle indicates the direction (red, positive correlation; blue, negative correlation) and strength of correlation. Only significantly correlations (*p* < 0.05) were shown.

We also correlated the relative abundance of different microbial genera and OTUs with the frequency of immune cell populations identified in the ileum and MLN. *Clostridium, Bacteroides*, and *Parabacteroides* were positively correlated with lymphoid cells frequency in the ileum (Figures 7A–C). Similarly, *Bacteroides, Bilophila, Clostridium, Parabacteroides, Phascolarctobacterium*, and *Ruminococcus* were positively correlated with monocyte/macrophage frequencies in the ileum, while *Butyricimonas* and *Bacteroides fragilis* exhibited negative correlation. Interestingly, the correlation of these bacterial genera with cDC was the opposite of that of monocytes/macrophages (Figures 7A–C). It remains to be determined how bacteria modulate the early development of the immune system in the gut. Future research using Gf piglets and the influential bacteria mentioned above can help address the question. As demonstrated in the present study, infants and Gf piglets may constitute a useful model to recapitulate early immune development.

Intestine is a critical site for mucosal and systemic immune system development and the gut microbiota plays fundamental roles in the induction, training, and function of the host immune system (66, 67). Previous studies in mice have shown that gut microbiota promotes cross-differentiation of naïve CD8^+^ T cells toward CD4^+^ T cells and controls postnatal development of invariant natural killer T cells (53, 68). *Enterobacteriaceae* in the gut promotes host IgA secretion, while *Bacteroides fragilis* and some *Clostridium* species enhance the activity of intestinal T-regulatory cells in mice (69–71). Despite these studies in rodent models, a comprehensive analysis of maturation of different mucosal lymphoid and myeloid immune cells in the gut-associated lymphoid tissues and intestines modulated by colonized diverse rural and urban human gut microbiota in any animal model system is lacking. Thus, the knowledge gained from this study will likely lead to investigations on the association of drastic changes in the frequency of important lymphoid and myeloid immune cell types to specific groups of bacteria and/or their metabolites in rural and urban infants. For this reason, we investigated how intestinal colonization with two different types of the fecal microbiome of infants (rural vs. urban) might impact the mucosal immune maturation in transplanted Gf piglets.

In conclusion, the gut microbiome of Amish infants differed markedly from that of non-Amish infants in both composition and structure, thus representing two types of distinct infant gut microbiomes (rural vs. urban). Using transplantation of diverse IFM to Gf piglets, we demonstrated that gut microbiome could differentially prime or modulate the early immune development in the gut. Furthermore, some commensal bacteria were strongly correlated with the frequencies of lymphoid and myeloid cells, which in turn lead to differential maturation of intestinal and mucosa-associated lymphoid tissues immune cells in the transplanted Gf piglets. Since we did not perform a side-by-side comparison of mice vs. pigs transplanted with the diverse HFM, it is difficult to conclude at this stage that pigs are better than mice as a model. However, so far, no studies have analyzed the influence of HFM of rural and urban infants on differential influence on mucosal immune system development happening at the intestines and mucosa-associated lymphoid tissues simultaneously in the humanized transplanted animal model. Our study in the Gf-piglet model provided that valuable missing insights, which will help in conducting in-depth studies to understand the implications of such early microbial diversity in precipitating allergic and metabolic diseases in children.

## Data Availability

All data generated or analyzed during this study are included in this published article (and its Supplementary Information files).

## Ethics Statement

This study involved infant fecal samples collection and the procedure of selection of human subjects was approved by the Institutional Review Board (2015H0281) of The Ohio State University. We confirm to share detailed protocols used in this manuscript upon request. The animal study was carried out in strict accordance with the recommendations of the Public Health Service Policy, United States Department of Agriculture Regulations, the National Research Council's Guide for the Care and Use of Laboratory Animals and the Federation of Animal Science Societies' Guide for the Care and Use of Agricultural Animals in Agricultural Research and Teaching. We followed all relevant institutional, state and federal regulations and policies regarding animal care and use at The Ohio State University. All the pigs were maintained, samples collected and euthanized per the protocols, and all the efforts were made to minimize the suffering of pigs. This study was carried out in accordance with the approved protocol of the Institutional Animal Care and Use Committee at The Ohio State University (Protocol number 2015A00000120).

## Author Contributions

SD, LW, ZY, and GR performed the experiments, analyzed the data, and drafted the manuscript. LA and JS performed the metagenomics study and analyzed the data. JR, PB, SG, KB, CS, YL, SR, BH, SK, MK, and JL involved in infant samples collection, processing of samples and performed animal experiments. All authors edited the manuscript and approved the final draft.

### Conflict of Interest Statement

The authors declare that the research was conducted in the absence of any commercial or financial relationships that could be construed as a potential conflict of interest.
