# Supplementary material for: Amish (Rural) vs. non-Amish (Urban) Infant Fecal Microbiotas Are Highly Diverse and Their Transplantation Lead to Differences in Mucosal Immune Maturation in a Humanized Germfree Piglet Model
Source: Front Immunol. 2019 Jul 16;10:1509. doi: 10.3389/fimmu.2019.01509 (PMC6648804; doi:10.3389/fimmu.2019.01509)
Supplement: Supplementary file 1 [file Table_1.DOCX]

**Abbreviations:**

**IFM: Infant fecal microbiota**

**RIFM: Rural-type (Amish) infant fecal microbiota**

**UIFM: Urban-type (non-Amish) infant fecal microbiota**

**RIFMP: Rural-type IFM transplanted piglets**

**UIFMP: Urban-type IFM transplanted piglets**

**OTUs: Operational taxonomic units**

**Table S1. Number of reads assigned after each step for different samples**

| **Sample Type** | **Group** | **Sample ID** | **Sequences assigned to an OTU** | **Sequences assigned to OTUs each representing >0.005% of total sequences** | **Sequences remained after removing chimeric sequences** | **Sequences after removing minor OTUs** |
| --- | --- | --- | --- | --- | --- | --- |
| **Feces** | Amish children | 1 | 29949 | 18399 | 16297 | 14875 |
|  |  | 2 | 32762 | 22175 | 19982 | 18864 |
|  |  | 3 | 37273 | 25782 | 23236 | 21830 |
|  |  | 4 | 30973 | 21239 | 18153 | 17377 |
|  |  | 5 | 37993 | 32499 | 27508 | 26565 |
|  | Non-Amish children | 1 | 27283 | 19508 | 17854 | 16675 |
|  |  | 2 | 33512 | 28111 | 23599 | 23329 |
|  |  | 3 | 34234 | 25860 | 23735 | 21897 |
|  |  | 4 | 34047 | 25596 | 22907 | 21415 |
|  |  | 5 | 47261 | 43419 | 35975 | 35213 |
| **Ileum Mucosa** | Amish/Rural IFM transplanted pigs (RIFMP) | 1 | 43376 | 41482 | 35622 | 34644 |
|  |  | 2 | 41680 | 40541 | 32224 | 31637 |
|  |  | 3 | 43990 | 42524 | 38658 | 37724 |
|  |  | 4 | 38675 | 36353 | 33315 | 32720 |
|  | Non-Amish/Urban IFM transplanted pigs (UIFMP) | 1 | 33345 | 31038 | 27788 | 27053 |
|  |  | 2 | 29421 | 28187 | 25158 | 24683 |
|  |  | 3 | 27723 | 24670 | 18256 | 17203 |
|  |  | 4 | 34477 | 32035 | 27678 | 26970 |
| **Ileum Digesta** | Amish/Rural IFM transplanted pigs (RIFMP) | 1 | 42495 | 40367 | 25552 | 24794 |
|  |  | 2 | 39932 | 37919 | 27680 | 27105 |
|  |  | 3 | 46155 | 44694 | 41681 | 41150 |
|  |  | 4 | 28854 | 27503 | 24804 | 24646 |
|  | Non-Amish/Urban IFM transplanted pigs (UIFMP) | 1 | 33425 | 27747 | 22375 | 21988 |
|  |  | 2 | 24464 | 22916 | 19524 | 19197 |
|  |  | 3 | 32422 | 29915 | 18031 | 17354 |
|  |  | 4 | 31380 | 28381 | 23132 | 22391 |
| **Colon Mucosa** | Amish/Rural IFM transplanted pigs (RIFMP) | 1 | 37389 | 33875 | 24685 | 23136 |
|  |  | 2 | 26122 | 22848 | 18886 | 17726 |
|  |  | 3 | 38869 | 35232 | 30446 | 29160 |
|  |  | 4 | 35525 | 33620 | 33084 | 30859 |
|  | Non-Amish/Urban IFM transplanted pigs (UIFMP) | 1 | 31024 | 27243 | 24446 | 22589 |
|  |  | 2 | 30960 | 26247 | 21393 | 19844 |
|  |  | 3 | 27187 | 23304 | 18300 | 16840 |
|  |  | 4 | 26306 | 21760 | 16222 | 14806 |
| **Colon Digesta** | Amish/Rural IFM transplanted pigs (RIFMP) | 1 | 30933 | 28140 | 21193 | 19583 |
|  |  | 2 | 33211 | 28383 | 24272 | 21509 |
|  |  | 3 | 38221 | 34501 | 27292 | 25442 |
|  |  | 4 | 35112 | 31340 | 27180 | 25230 |
|  | Non-Amish/Urban IFM transplanted pigs (UIFMP) | 1 | 18696 | 15353 | 13369 | 11751 |
|  |  | 2 | 22078 | 17916 | 12750 | 11693 |
|  |  | 3 | 23059 | 19467 | 14178 | 12698 |
|  |  | 4 | 31084 | 27083 | 20153 | 18323 |
| **Feces** | Amish/Rural IFM transplanted pigs (RIFMP) | 1 | 37714 | 32909 | 29323 | 26777 |
|  |  | 2 | 44724 | 42674 | 39616 | 37338 |
|  |  | 3 | 46289 | 44450 | 41230 | 39680 |
|  |  | 4 | 49258 | 45255 | 38299 | 35328 |
|  | Non-Amish/Urban IFM transplanted pigs (UIFMP) | 1 | 32429 | 28237 | 25110 | 23270 |
|  |  | 2 | 20185 | 16176 | 13444 | 11851 |
|  |  | 3 | 36747 | 33737 | 27993 | 26452 |
|  |  | 4 | 24206 | 20295 | 15837 | 14330 |

**Table S2a: Occurrence of major bacterial genera in the fecal microbiota of Amish or rural-type IFM donor (RIFM) and rural-type IFM transplanted piglets (RIFMP)***

| **Genus** | **RIFM** | **RIFMP** | | | | |
| --- | --- | --- | --- | --- | --- | --- |
|  |  | **Ileum mucosa** | **Ileum digesta** | **Colon mucosa** | **Colon digesta** | **Feces at necropsy** |
| *[Eubacterium]* | 0.068 (5) | 0.100 (4) | 0.002 (2) | 0.172 (4) | 0.104 (4) | 2.184 (4) |
| ***[Prevotella]*** | 0.371 (1) | 0.008 (1) |  | 0.028 (1) | 0.078 (1) |  |
| ***[Ruminococcus]*** | 0.733 (5) | 0.545 (4) | 0.014 (2) | 0.521 (4) | 0.985 (4) | 0.780 (4) |
| *Acinetobacter* |  | 0.105 (4) | 0.031 (4) | 0.003 (2) |  | 0.085 (4) |
| *Aerococcus* |  | 0.002 (1) |  |  | 0.001 (1) | 0.001 (1) |
| *Akkermansia* | 0.713 (3) | 0.370 (4) | 0.083 (1) | 13.435 (4) | 3.304 (4) | 5.888 (4) |
| *Anaerotruncus* | 0.013 (3) | 0.054 (4) | 0.010 (1) | 0.083 (4) | 0.351 (4) | 0.981 (4) |
| *Bacillus* |  | 0.002 (2) |  |  |  |  |
| ***Bacteroides*** | 26.724 (5) | 3.054 (4) | 0.267 (4) | 8.774 (4) | 16.757 (4) | 26.961 (4) |
| ***Bifidobacterium*** | 4.138 (5) | 0.189 (4) | 0.010 (3) | 0.047 (4) | 0.157 (4) | 0.228 (4) |
| *Bilophila* | 0.044 (2) | 0.134 (4) | 0.008 (2) | 0.557 (4) | 1.464 (4) | 0.032 (4) |
| *Blautia* | 1.851 (5) |  |  |  | 0.001 (1) |  |
| *Brevibacterium* |  | 0.015 (2) | 0.002 (1) | 0.003 (2) | 0.002 (1) |  |
| *Butyricimonas* | 0.113 (2) | 0.334 (4) | 0.008 (1) | 11.512 (4) | 12.563 (4) | 0.390 (4) |
| *Cellvibrio* |  |  | 0.002 (1) | 0.001 (1) |  | 0.070 (1) |
| *Christensenella* |  |  |  | 0.011 (2) | 0.017 (2) | 0.022 (3) |
| *Clostridium* | 0.064 2) | 10.667 (4) | 11.206 (4) | 0.765 (4) | 1.468 (4) | 0.475 (4) |
| *Corynebacterium* |  |  | 0.032 (4) | 0.006 (2) | 0.012 (4) | 0.002 (1) |
| *Delftia* |  | 0.001 (1) | 0.004 (1) |  |  | 0.006 (2) |
| *Desulfovibrio* | 0.054 (1) | 0.214 (4) | 0.007 (3) | 0.359 (4) | 0.371 (4) | 0.293 (4) |
| *Dorea* | 0.346 (3) | 0.009 (3) |  | 0.006 (3) | 0.025 (4) | 0.028 (4) |
| *Faecalibacterium* | 10.240 (4) |  |  |  |  |  |
| *Fimbriimonas* |  | 0.290 (4) | 0.125 (4) | 0.021 (4) | 0.005 (3) | 0.532 (4) |
| *Flavobacterium* |  |  | 0.002 (1) | 0.005 (2) |  |  |
| *Fusobacterium* | 0.035 (1) | 0.233 (4) | 0.004 (1) | 0.174 (4) | 0.228 (4) | 0.020 (3) |
| *Gemmata* |  | 0.049 (4) | 0.025 (3) | 0.003 (2) | 0.001 (1) | 0.031 (3) |
| *Haemophilus* | 0.394 (5) | 0.188 (4) | 0.164 (2) | 0.002 (2) |  | 0.002 (1) |
| *Halomonas* |  | 0.005 (3) | 0.001 (1) |  | 0.001 (1) | 0.012 (2) |
| *Lachnospira* | 1.452 (3) |  |  |  |  |  |
| *Lactobacillus* |  | 0.001 (2) | 0.004 (2) | 0.003 (1) | 0.003 (2) | 0.036 (2) |
| *Lactococcus* | 0.022 (3) | 0.079 (4) | 0.230 (4) | 0.178 (4) | 0.387 (4) | 0.040 (4) |
| *Megamonas* | 4.000 (4) |  |  |  |  |  |
| *Methylobacterium* |  |  |  |  |  | 0.020 (1) |
| *Ochrobactrum* |  | 0.001 (1) | 0.004 (2) | 0.001 (1) |  | 0.016 (2) |
| *Oscillospira* | 0.286 (5) | 0.155 (4) | 0.006 (2) | 0.372 (4) | 1.157 (4) | 0.571 (4) |
| ***Parabacteroides*** | 1.795 (4) | 0.890 (4) | 0.035 (3) | 3.658 (4) | 4.948 (4) | 3.000 (4) |
| *Perlucidibaca* |  | 0.305 (4) | 0.110 (4) | 0.023 (4) | 0.003 (3) | 0.555 (4) |
| *Phascolarctobacterium* | 0.260 (1) | 0.088 (4) | 0.008 (1) | 0.797 (4) | 0.655 (4) | 2.866 (4) |
| *Prevotella* | 24.863 (4) |  |  |  |  |  |
| *Propionibacterium* |  | 0.003 (2) | 0.002 (2) |  | 0.001 (1) | 0.001 (1) |
| *Proteus* |  | 0.002 (1) |  | 0.001 (1) |  |  |
| *Pseudomonas* |  |  |  |  |  | 0.001 (1) |
| *Pseudoramibacter_*  *Eubacterium* |  | 0.078 (4) | 0.005 (1) | 0.071 (4) | 0.421 (4) | 0.098 (4) |
| *Ramlibacter* |  |  |  |  |  | 0.012 (1) |
| *Roseburia* | 1.067 (5) | 0.001 (1) |  |  | 0.002 (1) | 0.012 (2) |
| *Rubrivivax* |  | 0.004 (2) | 0.008 (3) | 0.002 (2) |  | 0.023 (3) |
| *Ruminococcus* | 0.403 3) | 0.008 (2) |  |  |  |  |
| *Sediminibacterium* |  | 0.070 (4) | 0.022 (3) | 0.002 (1) | 0.001 (1) | 0.112 (4) |
| *Shewanella* |  | 0.347 (4) | 0.122 (4) | 0.006 (2) | 0.018 (3) | 1.231 (4) |
| *Staphylococcus* | 0.002 (2) | 0.172 (4) | 0.095 (4) | 0.071 (4) | 0.308 (4) | 0.034 (3) |
| *Streptococcus* | 0.747 (5) | 2.277 (4) | 2.134 (4) | 11.059 (4) | 5.512 (4) | 0.779 (4) |
| *Sutterella* | 0.800 (4) | 0.141 (4) | 0.003 (2) | 0.121 (4) | 0.230 (4) | 0.060 (3) |
| *Turicibacter* | 0.001 (1) | 12.849 (4) | 20.263 (4) | 0.107 (4) | 0.250 (3) | 0.457 (4) |
| *Veillonella* | 0.597 (4) | 0.167 (4) | 1.359 (4) |  |  |  |
| Total | 31 | 42 | 38 | 38 | 36 | 42 |

*The number in the parentheses indicated how many samples have this genus.

The bolded genera were relatively predominant in both the inoculum and the piglet gut samples, while the underlined genera were predominant in the inoculum but not found in the piglets.

**Table S2b. Occurrence of bacterial OTUs in the fecal microbiota of Amish or rural-type IFM donor (RIFM) and rural-type IFM transplanted piglets (RIFMP)***

| **#OTU ID** | **Taxon** | **RIFM** | **RIFMP** | | | | |
| --- | --- | --- | --- | --- | --- | --- | --- |
|  |  |  | **Ileum mucosa** | **Ileum digesta** | **Colon mucosa** | **Colon digesta** | **Feces at**  **necropsy** |
| OTU70 | c_*Betaproteobacteria* |  | 0.142 (4) | 0.032 (4) | 0.008 (3) | 0 | 0.142 (4) |
| OTU13 | o_*Acidimicrobiales* |  | 0.452 (4) | 0.216 (4) | 0.021 (4) | 0.006 (3) | 0.837 (4) |
| OTU155 | o_*Actinomycetales* |  | 0.003 (1) | 0.002 (2) |  |  |  |
| OTU148 | o_*Bacteroidales* |  | 0.027 (3) | 0.013 (2) | 0.002 (1) | 0.001 (1) | 0.057 (4) |
| OTU154 | o_*Clostridiales* | 0.001 (1) | 0.018 (3) | 0.001 (1) | 0.038 (4) | 0.167 (4) | 0.998 (4) |
| **OTU116** | **o_*Clostridiales*** | 0.384 (4) | 0.152 (4) | 0.146 (3) | 0.001 (1) | 0.001 (1) | 0.056 (2) |
| OTU149 | o_*Clostridiales* | 0.011 (3) | 0.004 (2) |  | 0.018 (4) | 0.080 (4) | 0.311 (4) |
| OTU156 | o_*Clostridiales* |  | 0.006 (3) | 0.008 (3) |  |  | 0.140 (4) |
| OTU251 | o_*Clostridiales* | 0.278 (1) | 0.003 (2) |  |  |  |  |
| OTU88 | o_*Clostridiales* | 3.701 (3) |  |  |  |  | 0.001 (1) |
| OTU208 | o_*Clostridiales* | 0.843 (3) |  |  |  |  |  |
| OTU256 | o_*Clostridiales* | 0.405 (3) |  |  |  |  |  |
| OTU57 | o_*Clostridiales* |  | 0.090 (4) | 0.034 (4) |  | 0.003 (2) | 0.732 (4) |
| OTU16 | o_*Ellin329* |  | 0.393 (4) | 0.166 (4) | 0.010 (3) | 0.007 (4) | 0.729 (4) |
| OTU41 | o_*Ellin6513* | 0.001 (1) | 0.183 (4) | 0.081 (4) | 0.010 (3) | 0.001 (1) | 0.332 (4) |
| OTU94 | o_*Myxococcales* |  | 0.041 (4) | 0.047 (4) | 0.008 (3) |  | 0.104 (4) |
| OTU98 | o_*Myxococcales* |  | 0.058 (4) | 0.013 (4) | 0.002 (1) | 0.001 (1) | 0.064 (4) |
| OTU105 | o_*Myxococcales* |  | 0.039 (4) | 0.020 (4) | 0.003 (1) | 0.003 (2) | 0.088 (4) |
| OTU260 | o_*RB41* |  |  |  |  | 0.001 (1) | 0.074 (1) |
| OTU268 | o_*RB41* |  |  |  |  |  | 0.002 (1) |
| OTU245 | o_*RF32* | 0.001 (1) | 0.043 (3) |  | 0.424 (4) | 0.039 (3) | 0.002 (1) |
| OTU220 | o_*RF32* | 0.001 (1) | 0.027 (4) | 0.005 (1) | 0.541 (4) | 0.147 (3) | 0.028 (3) |
| OTU26 | o_*Solibacterales* |  | 0.255 (4) | 0.112 (4) | 0.013 (3) | 0.010 (4) | 0.554 (4) |
| OTU74 | o_*Streptophyta* | 0.001 (1) | 0.045 (4) | 0.017 (4) |  | 0.004 (1) | 0.291 (3) |
| OTU240 | o_*Streptophyta* |  | 0.001 (1) |  |  |  | 0.005 (1) |
| **OTU40** | **f_*[Barnesiellaceae]*** | 0.289 (2) | 0.048 (4) | 0.008 (1) | 2.730 (4) | 1.408 (4) | 0.183 (4) |
| OTU185 | f_*[Barnesiellaceae]* | 0.014 (2) |  |  | 0.042 (3) | 0.072 (4) | 0.011 (1) |
| OTU221 | f_*[Barnesiellaceae]* | 0.026 (2) |  |  | 0.009 (2) | 0.003 (1) |  |
| OTU247 | f_*[Barnesiellaceae]* | 0.001 (1) |  |  | 0.276 (3) | 0.172 (4) |  |
| OTU50 | f_*[Barnesiellaceae]* |  |  |  |  | 0.001 (1) |  |
| OTU39 | f_*Bradyrhizobiaceae* |  | 0.180 (4) | 0.063 (3) | 0.008 (2) | 0.003 (1) | 0.209 (4) |
| OTU87 | f_*Bradyrhizobiaceae* |  | 0.073 (4) | 0.024 (3) | 0.004 (2) |  | 0.157 (3) |
| OTU115 | f_*Bradyrhizobiaceae* |  | 0.042 (4) | 0.012 (3) | 0.002 (1) | 0.001 (1) | 0.080 (4) |
| OTU86 | f_*Caulobacteraceae* |  | 0.075 (4) | 0.035 (3) | 0.002 (1) | 0.003 (2) | 0.095 (4) |
| OTU239 | f_*Caulobacteraceae* |  | 0.001 (2) | 0.010 (3) |  |  | 0.031 (2) |
| OTU2 | f_*Clostridiaceae* | 0.019 (4) | 18.497 (4) | 14.314 (4) | 0.048 (3) | 0.127 (3) | 0.119 (3) |
| OTU128 | f_*Clostridiaceae* | 0.017 (2) | 0.909 (4) | 0.698 (4) | 0.017 (2) | 0.009 (1) | 0.001 (1) |
| OTU147 | f_*Clostridiaceae* | 0.107 (4) | 0.127 (4) | 0.006 (2) |  |  | 0.045 (2) |
| **OTU1** | **f_*Enterobacteriaceae*** | **0.945 (5)** | 35.484 (4) | 40.462 (4) | 16.504 (4) | 23.074 (4) | 31.050 (4) |
| OTU28 | f_*Enterobacteriaceae* | 0.003 (1) | 0.697 (4) | 1.093 (4) | 2.807 (4) | 3.177 (4) | 0.285 (4) |
| OTU49 | f_*Enterobacteriaceae* | 0.111 (5) | 0.286 (4) | 1.012 (4) | 0.141 (4) | 0.259 (4) | 0.108 (4) |
| OTU101 | f_*Enterobacteriaceae* | 0.006 (1) | 0.050 (4) | 0.135 (4) | 0.288 (4) | 0.235 (4) | 0.035 (3) |
| OTU56 | f_*Enterobacteriaceae* |  | 0.006 (3) | 0.007 (3) | 0.001 (1) | 0.007 (4) | 0.003 (3) |
| OTU171 | f_*Erysipelotrichaceae* | 0.085 (4) | 0.008 (2) | 0.003 (1) | 0.008 (2) | 0.018 (3) | 0.291 (3) |
| OTU44 | f_*Isosphaeraceae* |  | 0.177 (4) | 0.064 (4) | 0.010 (4) | 0.003 (2) | 0.354 (4) |
| OTU32 | f_*Lachnospiraceae* | 0.119 (5) | 0.280 (4) | 0.012 (3) | 0.794 (4) | 1.313 (4) | 0.209 (4) |
| OTU146 | f_*Lachnospiraceae* | 0.001 (1) | 0.021 (4) |  | 0.103 (4) | 0.152 (4) | 0.036 (4) |
| OTU37 | f_*Lachnospiraceae* |  | 0.011 (4) | 0.002 (1) | 0.002 (2) |  | 1.177 (4) |
| OTU263 | f_*Lachnospiraceae* | 0.012 (1) | 0.004 (1) |  |  |  |  |
| OTU124 | f_*Lachnospiraceae* |  | 0.012 (4) | 0.007 (3) | 0.004 (1) | 0.004 (2) | 0.208 (4) |
| OTU212 | f_*Lachnospiraceae* | 0.082 (4) |  |  |  |  |  |
| OTU227 | f_*Lachnospiraceae* | 0.014 (3) |  |  |  | 0.001 (1) | 0.001 (1) |
| OTU280 | f_*Lachnospiraceae* | 0.001 (1) |  |  |  |  |  |
| OTU178 | f_*Lachnospiraceae* |  |  | 0.003 (1) | 0.003 (1) |  | 0.001 (1) |
| OTU175 | f_*Lachnospiraceae* | 0.020 (3) |  |  |  |  |  |
| OTU133 | f_*Lachnospiraceae* | 0.137 (1) |  |  | 0.002 (2) | 0.002 (2) | 0.005 (2) |
| OTU225 | f_*Lachnospiraceae* | 0.513 (3) |  |  |  |  |  |
| OTU188 | f_*Lachnospiraceae* | 0.404 (5) |  |  |  |  |  |
| OTU219 | f_*Lachnospiraceae* | 0.508 (4) |  |  |  |  |  |
| OTU169 | f_*Lachnospiraceae* | 0.486 (4) |  |  |  |  |  |
| OTU191 | f_*Lachnospiraceae* |  | 0.010 (3) | 0.004 (3) | 0.001 (1) |  | 0.032 (3) |
| OTU222 | f_*Lachnospiraceae* |  | 0.002 (1) |  |  | 0.001 (1) | 0.001 (2) |
| OTU270 | f_*Micrococcaceae* |  | 0.002 (1) |  |  |  |  |
| OTU182 | f_*mitochondria* |  | 0.035 (4) | 0.013 (1) | 0.001 (1) |  | 0.012 (1) |
| OTU237 | f_*Oxalobacteraceae* |  |  |  | 0.002 (1) |  |  |
| OTU55 | f_*Peptostreptococcaceae* | 0.006 (2) | 2.248 (4) | 2.526 (4) | 0.018 (3) | 0.015 (2) | 0.097 (3) |
| OTU233 | f_*Peptostreptococcaceae* |  | 0.091 (4) | 0.165 (4) |  | 0.001 (1) |  |
| OTU100 | f_*Peptostreptococcaceae* |  |  | 0.001 (1) |  |  |  |
| OTU136 | f_*Peptostreptococcaceae* | 0.001 (1) |  |  |  |  |  |
| OTU120 | f_*Planococcaceae* |  |  |  |  | 0.002 (2) |  |
| OTU78 | f_*Rhodospirillaceae* |  | 0.053 (4) | 0.032 (4) | 0.004 (2) | 0.001 (1) | 0.086 (4) |
| OTU112 | f_*Rhodospirillaceae* |  | 0.047 (4) | 0.013 (3) | 0.001 (1) |  | 0.064 (4) |
| OTU5 | f_*Rikenellaceae* |  | 1.044 (4) | 0.094 (3) | 9.308 (4) | 7.285 (4) | 1.177 (4) |
| **OTU17** | **f_*Rikenellaceae*** | 0.391 (3) | 0.380 (4) | 0.023 (1) | 5.141 (4) | 1.588 (4) | 0.674 (4) |
| OTU151 | f_*Ruminococcaceae* | 0.007 (1) | 0.246 (4) | 0.009 (3) | 0.749 (4) | 0.171 (4) | 0.016 (1) |
| OTU33 | f_*Ruminococcaceae* | 0.070 (4) | 0.081 (4) | 0.006 (3) | 0.151 (4) | 0.200 (4) | 1.520 (4) |
| OTU131 | f_*Ruminococcaceae* | 0.002 (1) | 0.029 (3) |  | 0.025 (3) | 0.026 (4) | 0.025 (3) |
| OTU165 | f_*Ruminococcaceae* |  | 0.012 (3) | 0.012 (3) |  |  | 0.046 (3) |
| OTU168 | f_*Ruminococcaceae* |  | 0.008 (4) | 0.005 (2) | 0.001 (1) | 0.001 (1) | 0.095 (4) |
| OTU203 | f_*Ruminococcaceae* |  | 0.010 (2) | 0.005 (2) |  | 0.003 (1) | 0.072 (3) |
| OTU257 | f_*Ruminococcaceae* | 0.290 (3) |  |  |  |  |  |
| OTU210 | f_*Ruminococcaceae* | 0.654 (4) |  |  |  |  |  |
| OTU241 | f_*Ruminococcaceae* | 0.360 (3) |  |  |  |  |  |
| OTU249 | f_*Ruminococcaceae* | 0.562 (2) |  |  |  |  |  |
| OTU275 | f_*Ruminococcaceae* |  |  |  |  | 0.001 (1) | 0.006 (2) |
| OTU194 | f_*S24-7* | 0.005 (1) | 0.058 (4) |  | 0.863 (4) | 0.156 (4) | 0.018 (3) |
| OTU261 | f_*S24-7* | 0.283 (1) | 0.022 (3) |  | 0.018 (2) | 0.007 (1) | 0.012 (1) |
| OTU66 | f_*Sinobacteraceae* |  | 0.119 (4) | 0.027 (3) | 0.005 (1) | 0.003 (3) | 0.128 (4) |
| OTU96 | f_*Sinobacteraceae* |  | 0.055 (4) | 0.011 (4) | 0.005 (2) |  | 0.190 (4) |
| OTU132 | f_*Sinobacteraceae* |  | 0.022 (4) | 0.012 (2) |  |  | 0.050 (4) |
| OTU97 | f_*Xanthomonadaceae* |  | 0.001 (1) |  |  |  |  |
| OTU253 | g_*[Prevotella]* | 0.371 (1) | 0.008 (1) |  | 0.028 (1) | 0.078 (1) |  |
| OTU61 | g_*[Ruminococcus]* | 0.072 (2) | 0.034 (4) |  | 0.056 (3) | 0.029 (3) | 0.508 (4) |
| OTU65 | g_*Acinetobacter* |  | 0.098 (4) | 0.029 (4) | 0.003 (2) |  | 0.062 (4) |
| OTU254 | g_*Acinetobacter* |  | 0.001 (1) | 0.001 (1) |  |  |  |
| OTU164 | g_*Acinetobacter* |  |  | 0.001 (1) |  |  |  |
| OTU173 | g_*Aerococcus* |  | 0.002 (1) |  |  | 0.001 (1) | 0.001 (1) |
| OTU92 | g_*Anaerotruncus* | 0.013 (3) | 0.054 (4) | 0.010 (1) | 0.083 (4) | 0.351 (4) | 0.980 (4) |
| OTU31 | g_*Bacteroides* | 0.118 (1) | 1.673 (4) | 0.038 (3) | 3.984 (4) | 2.385 (4) | 0.295 (4) |
| OTU60 | g_*Bacteroides* | 0.071 (5) | 0.192 (4) | 0.002 (2) | 0.220 (2) | 0.018 (3) | 0.157 (2) |
| **OTU62** | **g_*Bacteroides*** | 0.627 (3) | 0.071 (4) | 0.002 (1) | 0.321 (4) | 0.500 (4) | 0.095 (4) |
| **OTU15** | **g_*Bacteroides*** | 0.518 (3) | 0.156 (4) | 0.027 (2) | 1.128 (4) | 7.016 (4) | 3.311 (4) |
| **OTU6** | **g_*Bacteroides*** | 4.960 (5) | 0.106 (4) | 0.003 (2) | 0.269 (4) | 0.372 (4) | 1.678 (4) |
| OTU125 | g_*Bacteroides* | 0.051 (2) | 0.038 (3) | 0.001 (1) | 0.449 (4) | 0.763 (4) | 0.036 (4) |
| OTU123 | g_*Bacteroides* | 0.090 (3) | 0.009 (2) |  | 0.163 (3) | 0.558 (4) | 0.012 (3) |
| **OTU52** | **g_*Bacteroides*** | 0.722 (5) | 0.015 (2) |  | 0.063 (4) | 0.403 (4) | 0.032 (3) |
| OTU184 | g_*Bacteroides* | 0.006 (3) | 0.033 (4) | 0.002 (1) | 0.375 (4) | 0.246 (4) | 0.012 (2) |
| OTU198 | g_*Bacteroides* | 0.094 (4) | 0.004 (3) |  | 0.045 (3) | 0.167 (4) | 0.014 (2) |
| OTU111 | g_*Bacteroides* | 0.223 (5) | 0.004 (2) | 0.001 (1) | 0.019 (3) | 0.057 (4) | 0.191 (4) |
| OTU64 | g_*Bacteroides* | 0.410 (5) | 0.002 (2) |  | 0.019 (2) | 0.009 (1) | 0.065 (2) |
| OTU58 | g_*Bacteroides* | 0.162 (4) | 0.002 (2) |  | 0.067 (4) | 0.642 (4) | 0.434 (3) |
| OTU140 | g_*Bacteroides* | 0.126 (5) | 0.004 (2) |  | 0.002 (2) | 0.005 (2) | 0.004 (2) |
| OTU102 | g_*Bacteroides* | 0.714 (2) | 0.004 (2) |  | 0.032 (3) | 0.038 (2) |  |
| OTU72 | g_*Bacteroides* | 0.112 (4) | 0.001 (1) | 0.001 (1) | 0.057 (4) | 0.265 (4) | 0.454 (3) |
| OTU179 | g_*Bacteroides* | 0.028 (2) | 0.001 (1) |  |  |  |  |
| OTU205 | g_*Bacteroides* | 0.162 (1) |  |  | 0.001 (1) | 0.001 (1) |  |
| OTU202 | g_*Bacteroides* | 0.143 (3) |  |  |  |  |  |
| OTU142 | g_*Bacteroides* | 0.107 (3) |  |  | 0.010 (4) | 0.053 (4) | 0.013 (3) |
| OTU183 | g_*Bacteroides* | 0.114 (3) |  |  |  | 0.001 (1) | 0.002 (1) |
| OTU99 | g_*Bacteroides* | 0.179 (4) |  |  | 0.006 (3) | 0.033 (4) | 0.007 (2) |
| OTU107 | g_*Bacteroides* | 0.190 (5) |  |  | 0.004 (1) | 0.002 (1) | 0.019 (2) |
| OTU137 | g_*Bacteroides* | 0.102 (4) |  | 0.001 (1) | 0.007 (2) | 0.052 (4) | 0.194 (2) |
| OTU278 | g_*Bacteroides* | 0.069 (1) |  |  |  |  |  |
| OTU145 | g_*Bacteroides* | 0.136 (1) |  |  |  |  |  |
| OTU267 | g_*Bacteroides* | 0.042 (1) |  |  |  |  |  |
| OTU255 | g_*Bacteroides* | 0.015 (1) |  |  |  |  |  |
| OTU163 | g_*Bacteroides* | 0.027 (5) | 0.002 (1) |  | 0.024 (4) | 0.086 (3) | 0.352 (2) |
| OTU71 | g_*Bifidobacterium* | 0.120 (2) | 0.168 (4) | 0.002 (2) | 0.025 (4) | 0.053 (4) | 0.005 (3) |
| **OTU10** | **g_*Bifidobacterium*** | 2.044 (5) | 0.015 (3) | 0.002 (2) | 0.015 (2) | 0.088 (4) | 0.189 (3) |
| OTU42 | g_*Bifidobacterium* | 0.371 (4) | 0.001 (2) |  | 0.005 (2) | 0.003 (2) | 0.025 (2) |
| OTU143 | g_*Bifidobacterium* | 0.092 (4) | 0.001 (1) |  |  |  |  |
| OTU109 | g_*Bifidobacterium* | 0.076 (4) | 0.001 (1) | 0.001 (1) |  | 0.002 (2) | 0.002 (1) |
| OTU67 | g_*Bifidobacterium* | 0.179 (4) |  |  |  |  |  |
| OTU204 | g_*Bifidobacterium* | 0.042 (3) |  |  |  | 0.001 (1) | 0.001 (1) |
| OTU68 | g_*Bifidobacterium* | 0.241 (4) | 0.002 (2) | 0.005 (1) | 0.001 (1) | 0.008 (2) | 0.006 (2) |
| OTU76 | g_*Bilophila* | 0.044 (2) | 0.134 (4) | 0.008 (2) | 0.557 (4) | 1.464 (4) | 0.032 (4) |
| **OTU106** | **g_*Blautia*** | 1.552 (5) |  |  |  | 0.001 (1) |  |
| OTU197 | g_*Blautia* | 0.299 (5) |  |  |  |  |  |
| OTU215 | g_*Brevibacterium* |  | 0.0152 (2) | 0.002 (1) | 0.003 (2) | 0.002 (1) |  |
| OTU29 | g_*Butyricimonas* | 0.083 (1) | 0.197 (4) | 0.006 (1) | 7.323 (4) | 7.556 (4) | 0.225 (3) |
| OTU90 | g_*Butyricimonas* | 0.012 (2) | 0.084 (4) | 0.002 (1) | 2.017 (4) | 2.636 (4) | 0.087 (4) |
| OTU141 | g_*Butyricimonas* | 0.003 (2) | 0.048 (4) |  | 0.965 (4) | 1.057 (4) | 0.014 (2) |
| OTU117 | g_*Butyricimonas* | 0.015 (1) | 0.006 (1) |  | 1.207 (4) | 1.313 (4) | 0.064 (1) |
| OTU269 | g_*Cellvibrio* |  | 0.013 (1) | 0.002 (1) | 0.001 (1) |  | 0.070 (1) |
| OTU238 | g_*Christensenella* |  | 0.009 (2) |  | 0.006 (1) | 0.013 (1) | 0.017 (2) |
| OTU144 | g_*Christensenella* |  | 0.002 (1) |  | 0.004 (2) | 0.004 (2) | 0.004 (3) |
| OTU11 | g_*Clostridium* | 0.001 (1) | 10.574 (4) | 10.987 (4) | 0.752 (4) | 1.439 (4) | 0.472 (4) |
| OTU153 | g_*Clostridium* |  | 0.094 (4) | 0.219 (3) | 0.013 (3) | 0.029 (4) | 0.003 (1) |
| OTU192 | g_*Coprococcus* |  | 0.002 (2) |  |  |  |  |
| OTU157 | g_*Corynebacterium* |  | 0.022 (4) | 0.031 (3) | 0.006 (2) | 0.012 (4) | 0.002 (1) |
| OTU130 | g_*Corynebacterium* |  | 0.001 (1) |  |  |  |  |
| OTU236 | g_*Corynebacterium* |  |  | 0.001 (1) |  |  |  |
| OTU193 | g_*Delftia* |  | 0.001 (1) | 0.004 (1) |  |  | 0.006 (2) |
| OTU84 | g_*Desulfovibrio* | 0.054 (1) | 0.214 (4) | 0.007 (3) | 0.359 (4) | 0.371 (4) | 0.293 (4) |
| OTU152 | g_*Dorea* |  | 0.009 (3) |  | 0.006 (3) | 0.025 (4) | 0.028 (4) |
| OTU134 | g_*Dorea* | 0.346 (3) |  |  |  |  |  |
| OTU21 | g_*Fimbriimonas* |  | 0.254 (4) | 0.107 (4) | 0.017 (4) | 0.004 (3) | 0.449 (4) |
| OTU113 | g_*Fimbriimonas* |  | 0.036 (4) | 0.018 (4) | 0.004 (4) | 0.001 (1) | 0.083 (3) |
| OTU272 | g_*Flavobacterium* |  |  | 0.002 (1) |  |  |  |
| OTU104 | g_*Flavobacterium* |  |  |  | 0.002 (1) |  |  |
| OTU214 | g_*Flavobacterium* |  |  |  | 0.001 (1) |  |  |
| OTU242 | g_*Flavobacterium* |  |  |  | 0.001 (1) |  |  |
| OTU77 | g_*Fusobacterium* | 0.035 (1) | 0.233 (4) | 0.004 (1) | 0.174 (4) | 0.228 (4) | 0.020 (3) |
| OTU103 | g_*Gemmata* |  | 0.049 (4) | 0.025 (3) | 0.003 (2) | 0.001 (1) | 0.031 (3) |
| OTU20 | g_*Haemophilus* | 0.394 (5) | 0.188 (4) | 0.164 (2) | 0.002 (2) |  | 0.002 (1) |
| OTU201 | g_*Halomonas* |  | 0.005 (3) | 0.001 (1) |  | 0.001 (1) | 0.012 (2) |
| OTU232 | g_*Lachnospira* | 0.379 (2) |  |  |  |  |  |
| OTU150 | g_*Lachnospira* | 1.073 (2) |  |  |  |  |  |
| OTU224 | g_*Lactobacillus* |  |  | 0.001 (1) |  |  | 0.034 (2) |
| OTU82 | g_*Lactococcus* | 0.022 (3) | 0.079 (4) | 0.230 (4) | 0.178 (4) | 0.387 (4) | 0.040 (4) |
| OTU45 | g_*Megamonas* | 3.909 (4) |  |  |  |  |  |
| OTU262 | g_*Megamonas* | 0.091 (3) |  |  |  |  |  |
| OTU199 | g_*Methylobacterium* |  |  |  |  |  | 0.020 (1) |
| OTU38 | g_*Ochrobactrum* |  | 0.001 (1) | 0.004 (2) | 0.001 (1) |  | 0.016 (2) |
| OTU79 | g_*Oscillospira* | 0.256 (5) | 0.101 (4) | 0.004 (2) | 0.157 (4) | 0.566 (4) | 0.303 (4) |
| OTU172 | g_*Oscillospira* | 0.011 (1) | 0.028 (4) | 0.001 (1) | 0.065 (4) | 0.190 (4) | 0.045 (3) |
| OTU138 | g_*Oscillospira* | 0.018 (2) | 0.026 (2) | 0.001 (1) | 0.149 (3) | 0.401 (3) | 0.222 (4) |
| OTU95 | g_*Parabacteroides* |  | 0.242 (4) | 0.010 (1) | 1.382 (4) | 0.700 (4) | 0.371 (4) |
| OTU25 | g_*Parabacteroides* | 0.120 (2) | 0.152 (4) | 0.017 (2) | 1.015 (4) | 2.316 (4) | 1.315 (4) |
| OTU180 | g_*Parabacteroides* | 0.014 (2) |  |  | 0.005 (2) | 0.047 (3) | 0.015 (2) |
| OTU18 | g_*Perlucidibaca* |  | 0.305 (4) | 0.110 (4) | 0.023 (4) | 0.003 (3) | 0.555 (4) |
| OTU48 | g_*Phascolarctobacterium* | 0.260 (1) | 0.084 (4) | 0.005 (1) | 0.797 (4) | 0.655 (4) | 2.814 (4) |
| OTU213 | g_*Phascolarctobacterium* |  | 0.004 (1) | 0.003 (1) |  |  | 0.052 (3) |
| OTU35 | g_*Proteus* |  | 0.002 (1) |  | 0.001 (1) |  |  |
| OTU207 | g_*Pseudoramibacter_Eubacterium* |  | 0.078 (4) | 0.005 (1) | 0.071 (4) | 0.420 (4) | 0.098 (4) |
| OTU235 | g_*Ramlibacter* |  |  |  |  |  | 0.012 (1) |
| OTU244 | g_*Renibacterium* |  | 0.006 (2) |  |  |  |  |
| OTU196 | g_*Roseburia* | 1.067 (5) | 0.001 (1) |  |  | 0.002 (1) | 0.012 (2) |
| OTU186 | g_*Rubrivivax* |  | 0.004 (2) | 0.008 (3) | 0.002 (2) |  | 0.023 (3) |
| OTU69 | g_*Ruminococcus* |  | 0.008 (2) |  |  |  |  |
| OTU259 | g_*Ruminococcus* | 0.403 (3) |  |  |  |  |  |
| OTU81 | g_*Sediminibacterium* |  | 0.070 (4) | 0.022 (3) | 0.002 (1) | 0.001 (1) | 0.112 (4) |
| OTU7 | g_*Staphylococcus* | 0.002 (2) | 0.172 (4) | 0.094 (4) | 0.071 (4) | 0.308 (4) | 0.034 (3) |
| OTU8 | g_*Streptococcus* | 0.081 (3) | 2.164 (4) | 1.724 (4) | 10.840 (4) | 5.331 (4) | 0.773 (4) |
| OTU122 | g_*Streptococcus* | 0.001 (1) | 0.022 (3) | 0.039 (4) | 0.192 (3) | 0.110 (4) | 0.003 (2) |
| **OTU108** | **g_*Streptococcus*** | 0.624 (5) | 0.034 (4) | 0.043 (3) | 0.015 (3) | 0.026 (4) | 0.002 (1) |
| OTU121 | g_*Streptococcus* | 0.029 (3) | 0.056 (4) | 0.325 (3) | 0.010 (4) | 0.046 (3) |  |
| OTU126 | g_*Streptococcus* | 0.012 (4) |  | 0.001 (1) | 0.001 (1) |  | 0.001 (1) |
| **OTU83** | **g_*Sutterella*** | 0.794 (3) | 0.139 (4) | 0.003 (2) | 0.117 (3) | 0.225 (4) | 0.060 (3) |
| OTU200 | g_*Sutterella* |  | 0.002 (1) |  |  | 0.002 (1) |  |
| OTU231 | g_*Sutterella* | 0.007 (2) |  |  | 0.004 (3) | 0.003 (3) |  |
| OTU14 | g_*Turicibacter* |  | 12.364 (4) | 19.894 (4) | 0.105 (4) | 0.245 (3) | 0.052 (4) |
| OTU4 | g_*Turicibacter* | 0.001 (1) | 0.485 (4) | 0.369 (4) | 0.002 (2) | 0.004 (2) | 0.405 (4) |
| OTU174 | s_*[Eubacterium] dolichum* | 0.001 (1) | 0.089 (4) | 0.001 (1) | 0.160 (4) | 0.089 (4) | 0.822 (4) |
| OTU51 | s_*[Eubacterium] dolichum* | 0.066 (4) | 0.011 (3) | 0.001 (1) | 0.012 (2) | 0.014 (4) | 1.362 (4) |
| OTU89 | s_*[Ruminococcus] gnavus* |  | 0.510 (4) | 0.014 (2) | 0.463 (4) | 0.956 (4) | 0.268 (4) |
| OTU217 | s_*[Ruminococcus] gnavus* | 0.113 (5) | 0.001 (1) |  |  |  |  |
| OTU218 | s_*[Ruminococcus] gnavus* | 0.038 (3) |  |  |  |  |  |
| OTU43 | s_*[Ruminococcus] gnavus* | 0.511 (4) |  |  | 0.002 (2) |  | 0.005 (1) |
| OTU34 | s_*Acinetobacter rhizosphaerae* |  | 0.007 (1) |  |  |  | 0.022 (1) |
| **OTU12** | **s_*Akkermansia muciniphila*** | 0.688 (2) | 0.079 (4) |  | 5.296 (4) | 1.746 (3) | 5.351 (3) |
| OTU53 | s_*Akkermansia muciniphila* | 0.025 (1) | 0.290 (3) | 0.083 (1) | 8.139 (3) | 1.5589 (2) | 0.537 (3) |
| OTU54 | s_*Bacillus cereus* |  | 0.002 (2) |  |  |  |  |
| OTU119 | s_*Bacteroides acidifaciens* | 0.001 (1) | 0.003 (2) |  | 0.005 (2) | 0.036 (2) | 0.049 (2) |
| OTU158 | s_*Bacteroides caccae* | 0.029 (3) | 0.004 (3) |  | 0.062 (3) | 0.223 (4) | 0.087 (2) |
| OTU170 | s_*Bacteroides caccae* | 0.054 (3) |  |  | 0.010 (2) | 0.146 (3) | 0.003 (1) |
| **OTU3** | **s_*Bacteroides fragilis*** | 11.904 (5) | 0.345 (4) | 0.178 (3) | 0.745 (4) | 0.962 (4) | 18.213 (4) |
| OTU118 | s_*Bacteroides fragilis* | 0.270 (2) | 0.003 (2) | 0.001 (1) | 0.003 (3) | 0.005 (3) | 0.130 (4) |
| OTU75 | s_*Bacteroides ovatus* | 0.949 (4) | 0.019 (3) |  | 0.046 (4) | 0.287 (4) | 0.031 (4) |
| OTU176 | s_*Bacteroides ovatus* | 0.181 (3) | 0.002 (1) |  | 0.016 (3) | 0.071 (3) | 0.004 (1) |
| OTU27 | s_*Bacteroides plebeius* | 0.852 (1) |  |  |  |  |  |
| OTU159 | s_*Bacteroides plebeius* | 0.081 (1) |  |  |  |  |  |
| OTU206 | s_*Bacteroides plebeius* | 0.255 (1) |  |  |  |  |  |
| **OTU24** | **s_*Bacteroides uniformis*** | 1.471 (5) | 0.353 (4) | 0.011 (1) | 0.540 (4) | 1.252 (4) | 1.033 (4) |
| OTU110 | s_*Bacteroides uniformis* | 0.293 (4) | 0.011 (3) |  | 0.078 (2) | 0.080 (3) | 0.023 (2) |
| OTU216 | s_*Bacteroides uniformis* | 0.062 (2) |  |  | 0.002 (3) | 0.023 (4) | 0.009 (1) |
| OTU93 | s_*Bifidobacterium adolescentis* | 0.972 (4) |  |  |  | 0.002 (1) |  |
| OTU187 | s_*Clostridium neonatale* | 0.063 (1) |  |  |  |  |  |
| OTU63 | s_*Faecalibacterium prausnitzii* | 2.668 (4) |  |  |  |  |  |
| OTU80 | s_*Faecalibacterium prausnitzii* | 5.260 (4) |  |  |  |  |  |
| OTU226 | s_*Faecalibacterium prausnitzii* | 0.258 (4) |  |  |  |  |  |
| OTU127 | s_*Faecalibacterium prausnitzii* | 2.054 (4) |  |  |  |  |  |
| OTU266 | s_*Lactobacillus reuteri* |  | 0.001 (2) | 0.003 (1) | 0.003 (1) | 0.003 (2) | 0.002 (1) |
| **OTU36** | **s_*Parabacteroides distasonis*** | 0.482 (4) | 0.479 (4) | 0.009 (3) | 1.159 (4) | 1.552 (4) | 1.132 (4) |
| OTU177 | s_*Parabacteroides distasonis* | 0.087 (3) | 0.012 (3) |  | 0.012 (2) | 0.124 (3) | 0.066 (3) |
| OTU167 | s_*Parabacteroides distasonis* | 0.137 (4) | 0.002 (1) |  | 0.032 (4) | 0.109 (3) | 0.076 (2) |
| OTU46 | s_*Parabacteroides distasonis* | 0.950 (3) | 0.002 (2) |  | 0.003 (1) | 0.001 (1) | 0.002 (2) |
| OTU190 | s_*Parabacteroides distasonis* | 0.004 (2) | 0.001 (1) |  | 0.049 (3) | 0.099 (3) | 0.023 (2) |
| OTU19 | s_*Prevotella copri* | 12.915 (3) |  |  |  |  |  |
| OTU139 | s_*Prevotella copri* | 0.761 (3) |  |  |  |  |  |
| OTU189 | s_*Prevotella copri* | 0.639 (3) |  |  |  |  |  |
| OTU47 | s_*Prevotella copri* | 8.978 (4) |  |  |  |  |  |
| OTU181 | s_*Prevotella copri* | 1.570 (3) |  |  |  |  |  |
| OTU230 | s_*Propionibacterium acnes* |  | 0.003 (2) | 0.002 (2) |  | 0.001 (1) | 0.001 (1) |
| OTU271 | s_*Pseudomonas viridiflava* |  |  |  |  |  | 0.001 (1) |
| OTU209 | s_*Rothia mucilaginosa* | 0.009 (3) |  |  |  |  |  |
| OTU9 | s_*Shewanella algae* |  | 0.347 (4) | 0.122 (4) | 0.006 (2) | 0.018 (3) | 1.231 (4) |
| OTU129 | s_*Veillonella dispar* | 0.582 (4) | 0.113 (4) | 0.958 (4) |  |  |  |
| OTU211 | s_*Veillonella dispar* | 0.015 (1) | 0.053 (3) | 0.401 (4) |  |  |  |
| Total |  | 160 | 168 | 125 | 152 | 151 | 168 |

*The number in the parentheses indicated how many samples have this OTU.

The bolded OTUs were relatively predominant in both the inoculum and the piglet gut samples, while the underlined OTUs were predominant in the inoculum but not found in the piglets.

**Table S3a. Occurrence of major bacterial genera in the fecal microbiota of non-Amish or urban-type IFM donor (UIFM) and urban-type IFM transplanted piglets (UIFMP)**

| **Genus** | **UIFM** | **UIFMP** | | | | |
| --- | --- | --- | --- | --- | --- | --- |
|  |  | **Ileum mucosa** | **Ileum digesta** | **Colon mucosa** | **Colon digesta** | **Feces at necropsy** |
| *[Eubacterium]* | 0.068 (4) | 0.061 (4) | 0.026 (4) | 0.039 (4) | 0.027 (4) | 0.152 (4) |
| *[Ruminococcus]* | 0.451 (5) | 0.898 (4) | 0.004 (2) | 0.944 (4) | 0.992 (4) | 0.508 (4) |
| Acinetobacter |  | 0.002 (2) | 0.001 (1) |  |  |  |
| *Akkermansia* | 0.010 (2) | 5.301 (4) | 0.069 (3) | 6.620 (4) | 6.786 (4) | 16.474 (4) |
| *Anaerotruncus* | 0.025 (2) | 0.085 (4) | 0.004 (1) | 0.915 (4) | 0.160 (4) | 0.238 (4) |
| *Bacillus* |  | 0.002 (1) |  |  |  |  |
| ***Bacteroides*** | 51.691 (5) | 5.869 (4) | 0.211 (4) | 19.612 (4) | 18.824 (4) | 11.995 (4) |
| ***Bifidobacterium*** | 15.914 (5) | 0.212 (4) | 0.144 (3) | 0.114 (4) | 0.058 (3) | 0.086 (4) |
| *Bilophila* | 0.082 (1) | 0.427 (4) | 0.011 (1) | 0.564 (4) | 0.695 (4) | 0.486 (4) |
| *Blautia* | 1.361 (4) |  |  |  |  |  |
| *Butyricimonas* | 0.001 (1) | 0.224 (4) | 0.001 (1) | 0.568 (4) | 0.115 (4) | 0.495 (4) |
| *Christensenella* |  | 0.043 (4) |  | 0.041 (4) | 0.015 (3) | 0.072 (4) |
| *Clostridium* | 0.279 (3) | 9.972 (4) | 15.566 (4) | 0.470 (4) | 0.324 (5) | 0.132 (4) |
| *Corynebacterium* |  | 0.643 (4) | 0.113 (4) | 0.558 (4) | 0.403 (4) | 0.190 (4) |
| *Dorea* | 0.025 (3) | 0.007 (4) |  | 0.008 (3) | 0.009 (4) | 0.044 (3) |
| *Faecalibacterium* | 6.862 (4) |  |  |  |  |  |
| *Fimbriimonas* |  | 0.003 (2) |  |  |  |  |
| *Flavobacterium* |  | 0.001 (1) |  | 0.002 (1) |  |  |
| *Haemophilus* | 0.458 (5) | 0.001 (1) | 0.039 (2) | 0.004 (1) |  |  |
| *Halomonas* |  | 0.003 (2) |  |  |  |  |
| *Lachnospira* | 0.570 (4) |  |  |  |  |  |
| *Lactococcus* | 0.012 (1) | 0.276 (4) | 0.024 (4) | 0.189 (4) | 0.231 (4) | 0.331 (4) |
| *Megamonas* | 1.184 (2) |  |  |  |  |  |
| *Morganella* |  | 0.006 (3) | 0.334 (3) | 0.002 (2) | 0.002 (1) | 0.002 (1) |
| *Ochrobactrum* |  |  |  |  |  | 0.001 (1) |
| *Oscillospira* | 0.298 (4) | 0.887 (4) | 0.009 (2) | 1.142 (4) | 0.938 (4) | 1.071 (4) |
| ***Parabacteroides*** | 0.984 (3) | 5.650 (4) | 0.122 (3) | 8.021 (4) | 14.784 (4) | 10.790 (4) |
| *Perlucidibaca* |  | 0.008 (3) |  | 0.001 (1) |  |  |
| *Phascolarctobacterium* |  | 0.996 (4) | 0.005 (2) | 1.786 (4) | 2.618 (4) | 2.361 (4) |
| *Prevotella* | 6.799 (2) | 0.004 (2) |  | 0.001 (1) |  |  |
| *Proteus* |  | 0.264 (4) | 2.242 (4) | 0.019 (3) | 0.010 (3) | 0.020 (3) |
| *Roseburia* | 0.008 (1) |  |  |  |  |  |
| *Rothia* | 0.020 (3) |  |  |  |  |  |
| *Ruminococcus* | 0.164 (2) | 0.321 (4) | 0.001 (1) | 0.609 (4) | 1.003 (4) | 0.243 (4) |
| *Sediminibacterium* |  | 0.002 (1) |  |  |  |  |
| *Shewanella* |  | 0.065 (4) |  |  |  |  |
| *Staphylococcus* |  | 0.055 (4) | 0.022 (2) | 0.026 (4) | 0.032 (4) | 0.010 (3) |
| *Streptococcus* | 0.397 (5) | 5.723 (4) | 7.008 (4) | 4.945 (4) | 5.843 (4) | 5.044 (4) |
| ***Sutterella*** | 1.117 (5) | 0.680 (4) | 0.011 (1) | 1.349 (4) | 0.990 (4) | 0.492 (4) |
| *Turicibacter* | 0.006 (1) | 1.880 (4) | 6.347 (4) | 0.002 (2) | 0.004 (2) | 0.004 (3) |
| *Veillonella* | 0.306 (5) | 0.038 (4) | 0.076 (4) |  |  |  |
| *Wautersiella* |  | 0.002 (2) |  | 0.001 (1) |  |  |
| Total | 26 | 35 | 24 | 28 | 23 | 24 |

*The number in the parentheses indicated how many samples have this genus.

The bolded genera were relatively predominant in both the inoculum and the piglet gut samples, while the underlined genera were predominant in the inoculum but not found in the piglets.

**Table S3b. Occurrence of bacterial OTUs in the fecal microbiota of non-Amish or urban-type IFM donor (UIFM) and urban-type IFM transplanted piglets (UIFMP)***

| **#OTU ID** | **Taxon** | **UIFM** | **UIFMP** | | | | |
| --- | --- | --- | --- | --- | --- | --- | --- |
|  |  |  | **Ileum**  **mucosa** | **Ileum**  **digesta** | **Colon**  **mucosa** | **Colon**  **digesta** | **Feces at**  **necropsy** |
| OTU70 | c_*Betaproteobacteria* |  | 0.002 (2) |  |  |  |  |
| OTU13 | o_*Acidimicrobiales* |  | 0.012 (4) |  | 0.003 (1) |  |  |
| OTU73 | o_*Bacillales* |  | 0.001 (1) |  |  |  | 0.002 (1) |
| OTU88 | o_*Clostridiales* | 0.881 (2) |  |  |  |  |  |
| OTU208 | o_*Clostridiales* | 0.051 (3) |  |  |  |  |  |
| OTU116 | o_*Clostridiales* | 0.051 (2) | 0.943 (4) | 1.265 (4) | 0.003 (2) | 0.003 (2) |  |
| OTU251 | o_*Clostridiales* | 0.145 (1) |  |  |  |  |  |
| OTU57 | o_*Clostridiales* |  |  |  | 0.001 (1) |  |  |
| OTU16 | o_*Ellin329* |  | 0.009 (2) |  |  |  |  |
| OTU105 | o_*Myxococcales* |  | 0.001 (1) | 0.001 (1) |  |  |  |
| OTU94 | o_*Myxococcales* |  | 0.001 (1) |  | 0.002 (1) |  |  |
| OTU98 | o_*Myxococcales* |  | 0.001 (1) |  |  |  |  |
| OTU26 | o_*Solibacterales* |  | 0.008 (4) |  |  |  |  |
| OTU74 | o_*Streptophyta* | 0.001 (1) | 0.006 (3) |  |  |  |  |
| OTU40 | f_*[Barnesiellaceae]* | 0.001 (1) |  |  |  |  |  |
| OTU22 | f_*Bacillaceae* |  | 0.002 (1) | 0.001 (1) |  |  | 0.001 (1) |
| OTU39 | f_*Bradyrhizobiaceae* |  | 0.001 (1) |  |  |  |  |
| OTU86 | f_*Caulobacteraceae* |  | 0.003 (2) |  |  |  |  |
| OTU147 | f_*Clostridiaceae* | 0.001 (1) |  |  |  |  | 0.001 (1) |
| OTU2 | f_*Clostridiaceae* | 0.088 (3) | 5.678 (4) | 9.043 (4) | 0.017 (3) | 0.015 (2) | 0.013 (4) |
| OTU128 | f_*Clostridiaceae* | 0.017 (2) | 0.067 (3) | 0.027 (2) |  |  |  |
| **OTU1** | **f_*Enterobacteriaceae*** | 0.729 (5) | 21.839 (4) | 34.261 (4) | 1.734 (4) | 1.638 (4) | 6.001 (4) |
| OTU49 | f_*Enterobacteriaceae* | 0.141 (4) | 0.370 (4) | 1.134 (4) | 0.114 (4) | 0.083 (4) | 0.204 (4) |
| OTU101 | f_*Enterobacteriaceae* | 0.010 (1) | 0.110 (4) | 0.315 (4) | 0.0234 (4) | 0.016 (4) | 0.074 (4) |
| OTU28 | f_*Enterobacteriaceae* | 0.009 (1) | 1.169 (4) | 4.918 (4) | 0.732 (4) | 1.106 (4) | 1.395 (4) |
| OTU56 | f_*Enterobacteriaceae* | 0.096 (1) | 2.014 (4) | 3.932 (4) | 0.105 (4) | 0.110 (4) | 0.288 (3) |
| OTU135 | f_*Enterobacteriaceae* |  | 0.060 (3) | 0.805 (3) | 0.012 (4) | 0.031 (4) | 0.008 (3) |
| OTU171 | f_*Erysipelotrichaceae* | 0.341 (4) | 0.003 (2) | 0.001 (1) | 0.004 (2) | 0.004 (2) |  |
| OTU44 | f_*Isosphaeraceae* |  | 0.004 (1) |  |  |  |  |
| OTU225 | f_*Lachnospiraceae* | 0.119 (2) |  |  |  |  |  |
| OTU219 | f_*Lachnospiraceae* | 0.091 (2) |  |  |  |  |  |
| OTU169 | f_*Lachnospiraceae* | 0.218 (1) |  |  |  |  |  |
| OTU188 | f_*Lachnospiraceae* | 0.545 (3) |  |  |  |  |  |
| OTU133 | f_*Lachnospiraceae* | 0.001 (1) |  |  |  |  | 0.002 (1) |
| OTU32 | f_*Lachnospiraceae* | 0.379 (4) | 1.966 (4) | 0.030 (2) | 1.851 (4) | 2.164 (4) | 1.484 (4) |
| OTU212 | f_*Lachnospiraceae* | 0.014 (1) |  |  |  |  |  |
| OTU175 | f_*Lachnospiraceae* | 0.004 (1) |  |  |  |  |  |
| OTU227 | f_*Lachnospiraceae* | 0.504 (2) |  |  |  |  |  |
| OTU263 | f_*Lachnospiraceae* |  | 0.001 (1) |  |  |  |  |
| OTU280 | f_*Lachnospiraceae* | 0.259 (1) |  |  |  |  |  |
| OTU146 | f_*Lachnospiraceae* |  | 0.404 (4) | 0.004 (1) | 0.451 (4) | 0.322 (4) | 0.307 (4) |
| OTU252 | f_*Lachnospiraceae* | 0.421 (1) |  |  |  |  |  |
| OTU178 | f_*Lachnospiraceae* | 0.095 (2) | 0.309 (4) | 0.002 (1) | 0.241 (4) | 0.194 (4) | 0.114 (4) |
| OTU124 | f_*Lachnospiraceae* |  |  |  | 0.001 (1) |  |  |
| OTU222 | f_*Lachnospiraceae* | 0.005 (1) |  |  |  |  |  |
| OTU182 | f_*mitochondria* |  | 0.001 (1) |  |  |  |  |
| OTU237 | f_*Oxalobacteraceae* |  |  |  |  |  | 0.001 (1) |
| OTU55 | f_*Peptostreptococcaceae* | 0.012 (3) | 2.167 (3) | 2.670 (4) | 0.004 (3) |  |  |
| OTU136 | f_*Peptostreptococcaceae* | 0.002 (1) | 1.002 (4) | 2.084 (4) | 0.002 (2) | 0.004 (1) |  |
| OTU100 | f_*Peptostreptococcaceae* | 0.004 (1) | 1.682 (4) | 3.799 (4) | 0.001 (1) | 0.006 (2) |  |
| OTU233 | f_*Peptostreptococcaceae* |  | 0.104 (4) | 0.354 (3) |  |  |  |
| OTU120 | f_*Planococcaceae* |  | 0.003 (3) | 0.012 (1) | 0.002 (2) |  | 0.002 (1) |
| OTU112 | f_*Rhodospirillaceae* |  | 0.001 (1) |  |  |  |  |
| OTU17 | f_*Rikenellaceae* |  | 0.865 (4) | 0.014 (2) | 1.845 (4) | 1.210 (4) | 0.904 (4) |
| OTU5 | f_*Rikenellaceae* |  | 13.958 (4) | 0.340 (4) | 33.574 (4) | 26.936 (4) | 26.616 (4) |
| OTU210 | f_*Ruminococcaceae* | 0.119 (3) |  |  |  |  |  |
| OTU241 | f_*Ruminococcaceae* | 0.107 (1) |  |  |  |  |  |
| OTU257 | f_*Ruminococcaceae* | 0.140 (3) |  |  |  |  |  |
| OTU33 | f_*Ruminococcaceae* | 0.151 (3) | 1.016 (4) | 0.006 (2) | 2.488 (4) | 1.126 (4) | 2.609 (4) |
| OTU151 | f_*Ruminococcaceae* | 0.016 (1) |  |  |  |  |  |
| OTU131 | f_*Ruminococcaceae* |  | 0.388 (4) | 0.001 (1) | 0.348 (4) | 0.196 (4) | 0.183 (4) |
| OTU276 | f_*Ruminococcaceae* | 0.315 (2) |  |  |  |  |  |
| OTU203 | f_*Ruminococcaceae* |  |  |  | 0.001 (1) |  |  |
| OTU96 | f_*Sinobacteraceae* |  | 0.003 (1) |  |  |  |  |
| OTU66 | f_*Sinobacteraceae* |  | 0.001 (1) |  |  |  | 0.001 (1) |
| OTU61 | g_*[Ruminococcus]* | 0.094 (3) | 0.363 (4) |  | 0.370 (4) | 0.250 (3) | 0.222 (4) |
| OTU164 | g_*Acinetobacter* | 0.001 (1) |  |  |  |  |  |
| OTU65 | g_*Acinetobacter* |  | 0.002 (2) |  |  |  |  |
| OTU92 | g_*Anaerotruncus* | 0.025 (2) | 0.085 (4) | 0.004 (1) | 0.915 (4) | 0.160 (4) | 0.238 (4) |
| **OTU6** | **g_*Bacteroides*** | 28.042 (5) | 2.816 (4) | 0.098 (4) | 7.672 (4) | 9.683 (4) | 4.791 (4) |
| OTU52 | g_*Bacteroides* | 0.262 (4) | 0.018 (3) | 0.001 (1) | 0.063 (4) | 0.019 (3) | 0.031 (4) |
| **OTU102** | **g_*Bacteroides*** | 1.218 (1) | 0.511 (4) | 0.019 (1) | 1.050 (4) | 1.089 (4) | 0.743 (4) |
| OTU62 | g_*Bacteroides* |  | 0.274 (4) | 0.010 (1) | 0.559 (4) | 0.488 (4) | 0.270 (4) |
| **OTU15** | **g_*Bacteroides*** | 0.546 (1) | 0.768 (4) | 0.012 (1) | 2.199 (4) | 2.358 (4) | 1.478 (4) |
| **OTU64** | **g_*Bacteroides*** | 0.976 (5) | 0.016 (4) |  | 0.501 (4) | 0.181 (3) | 0.224 (4) |
| OTU111 | g_*Bacteroides* | 0.263 (2) | 0.006 (3) |  | 0.088 (4) | 0.012 (2) | 0.042 (4) |
| **OTU107** | **g_*Bacteroides*** | 0.580 (4) | 0.018 (4) |  | 0.493 (4) | 0.136 (3) | 0.191 (4) |
| **OTU99** | **g_*Bacteroides*** | 1.271 (4) | 0.048 (4) | 0.001 (1) | 0.342 (4) | 0.568 (4) | 0.216 (4) |
| OTU58 | g_*Bacteroides* | 0.234 (2) | 0.017 (3) |  | 0.420 (4) | 0.072 (3) | 0.247 (4) |
| OTU205 | g_*Bacteroides* | 0.472 (1) | 0.016 (4) | 0.003 (1) | 0.184 (4) | 0.316 (4) | 0.099 (4) |
| **OTU202** | **g_*Bacteroides*** | 0.544 (5) | 0.024 (3) | 0.001 (1) | 0.162 (4) | 0.321 (4) | 0.096 (4) |
| **OTU140** | **g_*Bacteroides*** | 0.609 (5) | 0.071 (4) | 0.012 (2) | 0.117 (4) | 0.130 (4) | 0.091 (4) |
| **OTU183** | **g_*Bacteroides*** | 0.779 (5) | 0.016 (3) |  | 0.117 (4) | 0.106 (4) | 0.066 (4) |
| OTU72 | g_*Bacteroides* | 0.117 (2) | 0.008 (3) | 0.002 (2) | 0.404 (4) | 0.073 (3) | 0.196 (4) |
| OTU142 | g_*Bacteroides* | 0.245 (2) | 0.039 (4) |  | 0.370 (4) | 0.603 (4) | 0.256 (4) |
| OTU137 | g_*Bacteroides* | 0.204 (4) | 0.008 (3) |  | 0.094 (4) | 0.010 (1) | 0.041 (4) |
| OTU198 | g_*Bacteroides* | 0.156 (2) | 0.010 (4) |  | 0.010 (4) | 0.011 (3) | 0.010 (3) |
| OTU123 | g_*Bacteroides* | 0.188 (1) | 0.041 (4) | 0.001 (1) | 0.341 (4) | 0.501 (4) | 0.224 (4) |
| OTU60 | *g_Bacteroides* | 0.771 (5) | 0.003 (2) |  | 0.176 (4) | 0.259 (4) | 0.125 (4) |
| OTU278 | g_*Bacteroides* | 0.215 (1) |  |  |  |  |  |
| OTU179 | g_*Bacteroides* | 0.398 (1) | 0.003 (1) |  | 0.032 (3) | 0.088 (3) | 0.011 (2) |
| OTU184 | g_*Bacteroides* |  | 0.011 (4) | 0.001 (1) | 0.083 (4) | 0.078 (4) | 0.043 (4) |
| OTU163 | g_*Bacteroides* | 0.009 (2) | 0.004 (2) |  | 0.044 (4) | 0.010 (1) | 0.012 (4) |
| **OTU10** | **g_*Bifidobacterium*** | 4.885 (5) | 0.108 (4) | 0.121 (3) | 0.043 (3) | 0.039 (2) | 0.045 (3) |
| OTU42 | g_*Bifidobacterium* | 4.317 (5) | 0.001 (1) |  | 0.001 (1) |  |  |
| OTU67 | g_*Bifidobacterium* | 2.171 (4) | 0.001 (1) |  |  |  |  |
| OTU71 | g_*Bifidobacterium* | 0.390 (2) | 0.090 (4) |  | 0.063 (4) | 0.007 (2) | 0.029 (3) |
| OTU143 | g_*Bifidobacterium* | 0.500 (4) | 0.001 (1) |  |  |  | 0.001 (1) |
| OTU109 | g_*Bifidobacterium* | 0.574 (5) | 0.001 (1) |  |  | 0.002 (1) |  |
| OTU204 | g_*Bifidobacterium* | 0.180 (5) |  | 0.001 (1) | 0.004 (3) |  | 0.002 (1) |
| **OTU68** | **g_*Bifidobacterium*** | 0.519 (4) | 0.010 (3) | 0.022 (2) | 0.004 (1) | 0.010 (1) | 0.009 (1) |
| OTU76 | g_*Bilophila* | 0.082 (1) | 0.427 (4) | 0.011 (1) | 0.564 (4) | 0.695 (4) | 0.486 (4) |
| OTU106 | g_*Blautia* | 0.982 (4) |  |  |  |  |  |
| OTU197 | g_*Blautia* | 0.379 (4) |  |  |  |  |  |
| OTU29 | g_*Butyricimonas* |  | 0.073 (4) |  | 0.147 (4) | 0.026 (3) | 0.149 (4) |
| OTU117 | g_*Butyricimonas* |  | 0.035 (4) |  | 0.192 (4) | 0.046 (4) | 0.166 (4) |
| OTU90 | g_*Butyricimonas* |  | 0.086 (4) |  | 0.168 (4) | 0.028 (4) | 0.130 (4) |
| OTU141 | g_*Butyricimonas* | 0.001 (1) | 0.029 (3) | 0.001 (1) | 0.061 (4) | 0.014 (2) | 0.050 (4) |
| OTU238 | g_*Christensenella* |  | 0.043 (4) |  | 0.041 (4) | 0.015 (3) | 0.072 (4) |
| OTU11 | g_*Clostridium* | 0.002 (1) | 9.784 (4) | 14.655 (4) | 0.457 (4) | 0.318 (4) | 0.124 (4) |
| OTU153 | g_*Clostridium* |  | 0.188 (4) | 0.909 (4) | 0.013 (4) | 0.006 (2) | 0.007 (2) |
| OTU23 | g_*Corynebacterium* |  | 0.493 (4) | 0.084 (4) | 0.413 (4) | 0.281 (4) | 0.122 (4) |
| OTU130 | g_*Corynebacterium* |  | 0.138 (4) | 0.029 (4) | 0.133 (4) | 0.120 (4) | 0.064 (4) |
| OTU234 | g_*Corynebacterium* |  | 0.010 (3) |  | 0.009 (3) | 0.002 (1) | 0.004 (1) |
| OTU236 | g_*Corynebacterium* |  | 0.001 (1) |  | 0.002 (1) |  |  |
| OTU134 | g_*Dorea* | 0.025 (3) | 0.001 (1) |  |  |  | 0.035 (2) |
| OTU152 | g_*Dorea* |  | 0.006 (4) |  | 0.008 (3) | 0.009 (4) | 0.008 (2) |
| OTU21 | g_*Fimbriimonas* |  | 0.003 (2) |  |  |  |  |
| OTU104 | g_*Flavobacterium* |  | 0.001 (1) |  | 0.002 (1) |  |  |
| OTU20 | g_*Haemophilus* | 0.458 (5) | 0.001 (1) | 0.038 (2) | 0.004 (1) |  |  |
| OTU201 | g_*Halomonas* |  | 0.003 (2) |  |  |  |  |
| OTU150 | g_*Lachnospira* | 0.330 (2) |  |  |  |  |  |
| OTU232 | g_*Lachnospira* | 0.240 (2) |  |  |  |  |  |
| OTU82 | g_*Lactococcus* | 0.012 (1) | 0.276 (4) | 0.024 (4) | 0.189 (4) | 0.231 (4) | 0.331 (4) |
| OTU45 | g_*Megamonas* | 1.114 (2) |  |  |  |  |  |
| OTU262 | g_*Megamonas* | 0.071 (2) |  |  |  |  |  |
| OTU274 | g_*Morganella* |  | 0.006 (3) | 0.334 (3) | 0.002 (2) | 0.002 (1) | 0.002 (1) |
| OTU38 | g_*Ochrobactrum* |  |  |  |  |  | 0.001 (1) |
| OTU79 | g_*Oscillospira* | 0.260 (4) | 0.392 (4) | 0.003 (2) | 0.668 (4) | 0.446 (4) | 0.719 (4) |
| OTU138 | g_*Oscillospira* | 0.020 (2) | 0.357 (4) | 0.003 (1) | 0.360 (4) | 0.323 (4) | 0.236 (4) |
| OTU172 | g_*Oscillospira* | 0.018 (2) | 0.138 (4) | 0.002 (1) | 0.114 (4) | 0.169 (4) | 0.116 (4) |
| OTU25 | g_*Parabacteroides* | 0.420 (2) | 2.510 (4) | 0.052 (3) | 3.504 (4) | 5.261 (4) | 3.512 (4) |
| OTU180 | g_*Parabacteroides* | 0.010 (1) | 0.128 (4) | 0.001 (1) | 0.320 (4) | 0.736 (4) | 0.520 (4) |
| OTU18 | g_*Perlucidibaca* |  | 0.008 (3) |  | 0.001 (1) |  |  |
| OTU48 | g_*Phascolarctobacterium* |  | 0.996 (4) | 0.005 (2) | 1.786 (4) | 2.618 (4) | 2.361 (4) |
| OTU250 | g_*Prevotella* | 0.591 (1) | 0.004 (2) |  | 0.001 (1) |  |  |
| OTU35 | g_*Proteus* |  | 0.264 (4) | 2.242 (4) | 0.019 (3) | 0.010 (3) | 0.020 (3) |
| OTU196 | g_*Roseburia* | 0.009 (1) |  |  |  |  |  |
| OTU259 | g_*Ruminococcus* | 0.065 (2) |  |  |  |  |  |
| OTU69 | g_*Ruminococcus* | 0.099 (1) | 0.321 (4) | 0.001 (1) | 0.609 (4) | 1.003 (4) | 0.243 (4) |
| OTU81 | g_*Sediminibacterium* |  | 0.002 (1) |  |  |  |  |
| OTU7 | g_*Staphylococcus* |  | 0.055 (4) | 0.022 (2) | 0.026 (4) | 0.032 (4) | 0.010 (3) |
| OTU108 | g_*Streptococcus* | 0.340 (4) | 0.008 (3) | 0.004 (2) |  |  |  |
| OTU8 | g_*Streptococcus* | 0.005 (3) | 5.337 (4) | 5.779 (4) | 4.619 (4) | 5.455 (4) | 4.694 (4) |
| OTU121 | g_*Streptococcus* | 0.022 (4) | 0.236 (4) | 0.715 (4) | 0.208 (4) | 0.219 (4) | 0.215 (4) |
| OTU126 | g_*Streptococcus* | 0.030 (3) | 0.004 (2) | 0.016 (2) |  |  | 0.003 (2) |
| OTU122 | g_*Streptococcus* |  | 0.138 (4) | 0.495 (4) | 0.118 (4) | 0.169 (3) | 0.132 (4) |
| **OTU83** | **g_*Sutterella*** | 0.552 (3) | 0.560 (4) | 0.009 (1) | 1.060 (4) | 0.855 (4) | 0.394 (4) |
| OTU231 | g_*Sutterella* | 0.059 (1) | 0.006 (2) |  | 0.006 (2) | 0.012 (3) | 0.007 (2) |
| **OTU200** | **g_*Sutterella*** | 0.506 (1) | 0.113 (4) | 0.002 (1) | 0.283 (4) | 0.123 (4) | 0.091 (4) |
| OTU4 | g_*Turicibacter* | 0.006 (1) | 0.034 (3) | 0.123 (3) |  |  | 0.003 (2) |
| OTU14 | g_*Turicibacter* |  | 1.846 (4) | 6.224 (4) | 0.002 (2) | 0.004 (2) | 0.002 (1) |
| OTU59 | g_*Veillonella* | 0.001 (1) |  | 0.002 (1) |  |  |  |
| OTU30 | g_*Wautersiella* |  | 0.002 (2) |  |  |  |  |
| OTU195 | g_*Wautersiella* |  |  |  | 0.001 (1) |  |  |
| OTU51 | s_*[Eubacterium] dolichum* | 0.068 (4) | 0.061 (4) | 0.026 (4) | 0.039 (4) | 0.027 (4) | 0.150 (4) |
| OTU174 | s_*[Eubacterium] dolichum* |  |  |  |  |  | 0.002 (1) |
| OTU43 | s_*[Ruminococcus] gnavus* | 0.321 (4) | 0.148 (4) | 0.001 (1) | 0.165 (4) | 0.130 (4) | 0.112 (4) |
| OTU217 | s_*[Ruminococcus] gnavus* | 0.003 (2) | 0.001 (1) |  | 0.001 (1) |  |  |
| OTU218 | s_*[Ruminococcus] gnavus* | 0.023 (3) | 0.008 (1) |  | 0.006 (2) | 0.015 (3) | 0.004 (1) |
| OTU89 | s_*[Ruminococcus] gnavus* | 0.010 (1) | 0.378 (4) | 0.003 (2) | 0.402 (4) | 0.597 (4) | 0.170 (4) |
| OTU34 | s_*Acinetobacter rhizosphaerae* |  |  | 0.001 (1) |  |  |  |
| OTU12 | s_*Akkermansia muciniphila* | 0.008 (2) | 5.301 (4) | 0.068 (3) | 6.620 (4) | 6.784 (4) | 16.474 (4) |
| OTU53 | s_*Akkermansia muciniphila* | 0.001 (1) |  |  |  | 0.002 (1) |  |
| OTU54 | s_*Bacillus cereus* |  | 0.002 (1) |  |  |  |  |
| OTU170 | s_*Bacteroides caccae* | 0.072 (1) | 0.001 (1) |  | 0.023 (4) | 0.007 (3) | 0.003 (2) |
| OTU158 | s_*Bacteroides caccae* | 0.010 (1) | 0.022 (4) |  | 0.123 (4) | 0.191 (4) | 0.151 (4) |
| **OTU3** | **s_*Bacteroides fragilis*** | 2.304 (5) | 0.479 (4) | 0.018 (2) | 2.686 (4) | 0.404 (4) | 1.540 (4) |
| OTU118 | s_*Bacteroides fragilis* |  |  |  |  |  | 0.005 (2) |
| OTU265 | s_*Bacteroides fragilis* |  |  |  | 0.010 (2) | 0.006 (1) | 0.002 (1) |
| OTU75 | s_*Bacteroides ovatus* | 0.290 (2) | 0.008 (3) | 0.007 (1) | 0.030 (3) | 0.012 (2) | 0.010 (2) |
| OTU176 | s_*Bacteroides ovatus* | 0.067 (1) |  | 0.001 (1) | 0.006 (3) | 0.007 (1) | 0.004 (2) |
| **OTU27** | **s_*Bacteroides plebeius*** | 0.994 (1) | 0.130 (4) | 0.013 (1) | 0.195 (3) | 0.271 (3) | 0.085 (3) |
| OTU206 | s_*Bacteroides plebeius* | 0.045 (1) | 0.004 (1) |  | 0.025 (3) | 0.038 (3) | 0.006 (3) |
| OTU159 | s_*Bacteroides plebeius* | 0.436 (1) | 0.004 (1) |  | 0.050 (3) | 0.076 (3) | 0.014 (2) |
| **OTU24** | **s_*Bacteroides uniformis*** | 7.611 (5) | 0.456 (4) | 0.008 (2) | 0.695 (4) | 0.532 (4) | 0.530 (4) |
| **OTU110** | **s_*Bacteroides uniformis*** | 1.737 (4) | 0.013 (4) |  | 0.161 (4) | 0.145 (4) | 0.096 (4) |
| OTU216 | s_*Bacteroides uniformis* | 0.026 (3) | 0.004 (2) |  | 0.084 (4) | 0.021 (4) | 0.046 (4) |
| OTU93 | s_*Bifidobacterium adolescentis* | 2.378 (5) |  |  |  |  |  |
| OTU187 | s_*Clostridium neonatale* | 0.277 (3) |  | 0.002 (1) |  |  |  |
| OTU80 | s_*Faecalibacterium prausnitzii* | 0.804 (4) |  |  |  |  |  |
| OTU63 | s_*Faecalibacterium prausnitzii* | 5.103 (3) |  |  |  |  |  |
| OTU127 | s_*Faecalibacterium prausnitzii* | 0.541 (3) |  |  |  |  |  |
| OTU226 | s_*Faecalibacterium prausnitzii* | 0.414 (4) |  |  |  |  |  |
| OTU46 | s_*Parabacteroides distasonis* | 0.019 (3) | 1.866 (4) | 0.051 (3) | 2.598 (4) | 5.326 (4) | 4.164 (4) |
| OTU36 | s_*Parabacteroides distasonis* | 0.217 (2) | 0.908 (4) | 0.014 (1) | 1.102 (4) | 2.487 (4) | 1.798 (4) |
| OTU167 | s_*Parabacteroides distasonis* | 0.021 (2) | 0.021 (4) |  | 0.130 (4) | 0.235 (4) | 0.194 (4) |
| OTU177 | s_*Parabacteroides distasonis* | 0.274 (2) | 0.122 (4) | 0.001 (1) | 0.129 (4) | 0.160 (4) | 0.157 (4) |
| OTU190 | s_*Parabacteroides distasonis* | 0.023 (1) | 0.096 (4) | 0.002 (1) | 0.237 (4) | 0.578 (4) | 0.443 (4) |
| OTU19 | s_*Prevotella copri* | 4.789 (2) |  |  |  |  |  |
| OTU47 | s_*Prevotella copri* | 0.112 (1) |  |  |  |  |  |
| OTU181 | s_*Prevotella copri* | 0.001 (1) |  |  |  |  |  |
| OTU139 | s_*Prevotella copri* | 1.006 (1) |  |  |  |  |  |
| OTU189 | s_*Prevotella copri* | 0.299 (1) |  |  |  |  |  |
| OTU209 | s_*Rothia mucilaginosa* | 0.020 (3) |  |  |  |  |  |
| OTU9 | s_*Shewanella algae* |  | 0.065 (4) |  |  |  |  |
| OTU129 | s_*Veillonella dispar* | 0.284 (5) | 0.029 (4) | 0.050 (4) |  |  |  |
| OTU211 | s_*Veillonella dispar* | 0.020 (2) | 0.009 (1) | 0.024 (2) |  |  |  |
| Total |  | 137 | 141 | 85 | 114 | 100 | 109 |

*The number in the parentheses indicated how many samples have this OTU.

The bolded OTUs were relatively predominant in both the inoculum and the piglet gut samples, while the underlined OTUs were predominant in the inoculum but not found in the piglets.

Table S4. Summary of the OTUs with significant difference (based on LEfSe) in relative abundance between the rural and the urban samples*

| OTU ID | Taxa | Infant’s fecal inoculum | Humanized piglets | | | | | |
| --- | --- | --- | --- | --- | --- | --- | --- | --- |
|  |  |  | Ileum | | Colon | | Feces | |
|  |  |  | IM | ID | CM | CD | FS1 | FSN |
| 1 | f_Enterobacteriaceae |  |  |  |  | R>U |  | R>U |
| 3 | s_Bacteroides fragilis |  |  |  |  |  | R>U | R>U |
| 4 | g_Turicibacter |  |  |  |  |  |  | R>U |
| 5 | f_Rikenellaceae |  | U>R |  | U>R | U>R |  | U>R |
| 6 | g_Bacteroides | U>R | U>R | U>R | U>R | U>R |  |  |
| 7 | g_Staphylococcus |  |  |  |  | R>U |  |  |
| 8 | g_Streptococcus |  |  |  |  |  |  | U>R |
| 9 | s_Shewanellaalgae |  |  | R>U |  | R>U |  | R>U |
| 10 | g_Bifidobacterium |  | U>R |  |  |  |  |  |
| 12 | s_Akkermansia muciniphila |  | U>R | U>R |  |  |  |  |
| 13 | o_Acidimicrobiales |  | R>U | R>U |  | R>U |  | R>U |
| 14 | g_Turicibacter |  |  |  | R>U |  |  | R>U |
| 15 | g_Bacteroides |  | U>R |  |  |  |  |  |
| 16 | o_Ellin329 |  | R>U | R>U | R>U | R>U |  | R>U |
| 17 | f_Rikenellaceae |  |  |  | R>U |  |  |  |
| 18 | g_Perlucidibaca |  | R>U | R>U | R>U | R>U |  | R>U |
| 20 | g_Haemophilus |  | R>U |  |  |  |  |  |
| 21 | g_Fimbriimonas |  | R>U | R>U | R>U | R>U |  | R>U |
| 23 | g_Corynebacterium |  | U>R | U>R | U>R | U>R |  | U>R |
| 25 | g_Parabacteroides |  | U>R |  | U>R |  | U>R |  |
| 26 | o_Solibacterales |  | R>U | R>U | R>U | R>U |  | R>U |
| 27 | s_Bacteroidesplebeius |  | U>R |  | U>R | U>R |  | U>R |
| 28 | f_Enterobacteriaceae |  |  |  |  | R>U |  | U>R |
| 29 | g_Butyricimonas |  | R>U |  | R>U | R>U |  |  |
| 31 | g_Bacteroides |  | R>U | R>U | R>U | R>U | R>U | R>U |
| 32 | f_Lachnospiraceae |  | U>R |  |  |  |  |  |
| 33 | f_Ruminococcaceae |  | U>R |  | U>R | U>R |  |  |
| 35 | g_Proteus |  | U>R | U>R |  | U>R |  | U>R |
| 37 | f_Lachnospiraceae |  | R>U |  |  |  |  | R>U |
| 39 | f_Bradyrhizobiaceae |  | R>U | R>U |  |  |  | R>U |
| 40 | f_[Barnesiellaceae] |  | R>U |  | R>U | R>U | R>U | R>U |
| 41 | o_Ellin6513 |  | R>U | R>U | R>U |  |  | R>U |
| 43 | s_[Ruminococcus] gnavus |  | U>R |  | U>R | U>R |  | U>R |
| 44 | f_Isosphaeraceae |  | R>U | R>U | R>U |  |  | R>U |
| 46 | s_Parabacteroides distasonis |  | U>R | U>R | U>R | U>R | R>U | U>R |
| 48 | g_Phascolarctobacterium |  | U>R |  |  | U>R | R>U |  |
| 49 | f_Enterobacteriaceae |  |  |  |  | R>U |  |  |
| 51 | s_[Eubacterium] dolichum |  | U>R | U>R |  |  |  |  |
| 52 | g_Bacteroides |  |  |  |  | R>U |  |  |
| 53 | s_Akkermansia muciniphila |  | R>U |  | R>U |  | R>U | R>U |
| 55 | f_Peptostreptococcaceae |  |  |  |  |  |  | R>U |
| 56 | f_Enterobacteriaceae |  | U>R | U>R | U>R | U>R | U>R |  |
| 57 | o_Clostridiales |  | R>U | R>U |  |  |  | R>U |
| 58 | g_Bacteroides |  |  |  |  |  | R>U |  |
| 64 | g_Bacteroides |  | U>R |  | U>R |  |  | U>R |
| 65 | g_Acinetobacter |  | R>U | R>U |  |  |  | R>U |
| 66 | f_Sinobacteraceae |  | R>U | R>U |  | R>U |  | R>U |
| 69 | g_Ruminococcus |  | U>R |  | U>R | U>R |  | U>R |
| 70 | c_Betaproteobacteria |  | R>U | R>U | R>U |  |  | R>U |
| 71 | g_Bifidobacterium |  |  |  |  | R>U |  |  |
| 72 | g_Bacteroides |  |  |  | U>R |  | R>U |  |
| 74 | o_Streptophyta |  | R>U | R>U |  |  |  | R>U |
| 76 | g_Bilophila |  |  |  |  |  |  | U>R |
| 77 | g_Fusobacterium |  | R>U |  | R>U | R>U | R>U | R>U |
| 78 | f_Rhodospirillaceae |  | R>U | R>U |  |  |  | R>U |
| 79 | g_Oscillospira |  | U>R |  |  |  | R>U |  |
| 81 | g_Sediminibacterium |  | R>U | R>U |  |  |  | R>U |
| 83 | g_Sutterella |  |  |  | U>R |  |  | U>R |
| 84 | g_Desulfovibrio |  | R>U | R>U | R>U | R>U |  | R>U |
| 86 | f_Caulobacteraceae |  | R>U | R>U |  |  |  | R>U |
| 87 | f_Bradyrhizobiaceae |  | R>U | R>U |  |  |  | R>U |
| 90 | g_Butyricimonas |  |  |  |  | R>U |  |  |
| 94 | o_Myxococcales |  | R>U | R>U |  |  |  | R>U |
| 95 | g_Parabacteroides |  | R>U |  | R>U | R>U |  | R>U |
| 96 | f_Sinobacteraceae |  | R>U | R>U |  |  |  | R>U |
| 98 | o_Myxococcales |  | R>U | R>U |  |  |  | R>U |
| 99 | g_Bacteroides |  | U>R |  | U>R |  |  | U>R |
| 100 | f_Peptostreptococcaceae |  | U>R | U>R |  |  |  |  |
| 101 | f_Enterobacteriaceae |  |  |  |  | R>U |  |  |
| 102 | g_Bacteroides |  | U>R |  | U>R | U>R |  | U>R |
| 103 | g_Gemmata |  | R>U | R>U |  |  |  | R>U |
| 105 | o_Myxococcales |  | R>U | R>U |  |  |  | R>U |
| 107 | g_Bacteroides |  | U>R |  | U>R |  |  | U>R |
| 108 | g_Streptococcus |  |  |  | R>U | R>U |  |  |
| 110 | s_Bacteroides uniformis |  |  |  |  |  |  | U>R |
| 112 | f_Rhodospirillaceae |  | R>U | R>U |  |  |  | R>U |
| 113 | g_Fimbriimonas |  | R>U | R>U | R>U |  |  | R>U |
| 115 | f_Bradyrhizobiaceae |  | R>U | R>U |  |  |  | R>U |
| 117 | g_Butyricimonas |  |  |  |  | R>U |  |  |
| 118 | s_Bacteroides fragilis |  |  |  | R>U | R>U | R>U |  |
| 120 | f_Planococcaceae |  | U>R |  |  |  |  |  |
| 121 | g_Streptococcus |  | U>R |  | U>R |  |  | U>R |
| 122 | g_Streptococcus |  |  |  |  |  |  | U>R |
| 123 | g_Bacteroides |  |  |  |  |  |  | U>R |
| 124 | f_Lachnospiraceae |  | R>U | R>U |  |  |  | R>U |
| 125 | g_Bacteroides |  | R>U |  | R>U | R>U | R>U | R>U |
| 130 | g_Corynebacterium |  | U>R | U>R | U>R | U>R |  | U>R |
| 131 | f_Ruminococcaceae |  |  |  |  |  |  | U>R |
| 132 | f_Sinobacteraceae |  | R>U |  |  |  |  | R>U |
| 135 | f_Enterobacteriaceae |  | U>R | U>R | U>R | U>R |  | U>R |
| 136 | f_Peptostreptococcaceae |  | U>R | U>R |  |  |  |  |
| 137 | g_Bacteroides |  | U>R |  | U>R |  |  |  |
| 138 | g_Oscillospira |  | U>R |  |  |  |  |  |
| 140 | g_Bacteroides | U>R | U>R |  | U>R | U>R |  | U>R |
| 141 | g_Butyricimonas |  |  |  |  | R>U |  |  |
| 142 | g_Bacteroides |  | U>R |  | U>R | U>R |  | U>R |
| 144 | g_Christensenella |  |  |  |  |  |  | R>U |
| 146 | f_Lachnospiraceae |  | U>R |  |  |  |  | U>R |
| 147 | f_Clostridiaceae |  | R>U |  |  |  |  |  |
| 148 | o_Bacteroidales |  | R>U |  |  |  |  | R>U |
| 149 | o_Clostridiales |  |  |  | R>U | R>U |  | R>U |
| 151 | f_Ruminococcaceae |  | R>U | R>U | R>U | R>U | U>R |  |
| 152 | g_Dorea |  |  |  |  |  | R>U |  |
| 153 | g_Clostridium |  |  | U>R |  | R>U |  |  |
| 154 | o_Clostridiales |  | R>U |  | R>U | R>U |  | R>U |
| 156 | o_Clostridiales |  | R>U | R>U |  |  |  | R>U |
| 157 | g_Corynebacterium |  | R>U | R>U |  | R>U |  |  |
| 159 | s_Bacteroides plebeius |  |  |  | U>R | U>R |  |  |
| 163 | g_Bacteroides | R>U |  |  |  |  | R>U |  |
| 165 | f_Ruminococcaceae |  | R>U | R>U |  |  |  | R>U |
| 167 | s_Parabacteroides distasonis |  | U>R |  | U>R |  |  |  |
| 168 | f_Ruminococcaceae |  | R>U |  |  |  |  | R>U |
| 171 | f_Erysipelotrichaceae |  |  |  |  |  |  | R>U |
| 172 | g_Oscillospira |  | U>R |  |  |  |  |  |
| 174 | s_[Eubacterium] dolichum |  | R>U |  | R>U | R>U |  | R>U |
| 177 | s_Parabacteroides distasonis |  | U>R |  |  |  |  |  |
| 178 | f_Lachnospiraceae |  | U>R |  | U>R | U>R | U>R | U>R |
| 179 | g_Bacteroides |  |  |  | U>R | U>R |  |  |
| 180 | g_Parabacteroides |  | U>R |  | U>R | U>R |  | U>R |
| 182 | f_mitochondria |  | R>U |  |  |  |  |  |
| 183 | g_Bacteroides |  | U>R |  | U>R | U>R |  | U>R |
| 185 | f_[Barnesiellaceae] |  |  |  | R>U | R>U |  |  |
| 186 | g_Rubrivivax |  |  | R>U |  |  |  | R>U |
| 190 | s_Parabacteroides distasonis |  | U>R |  | U>R | U>R |  | U>R |
| 191 | f_Lachnospiraceae |  | R>U | R>U |  |  |  | R>U |
| 194 | f_S24U>R7 |  | R>U |  | R>U | R>U |  | R>U |
| 196 | g_Roseburia | R>U |  |  |  |  |  |  |
| 200 | g_Sutterella |  | U>R |  | U>R | U>R |  | U>R |
| 202 | g_Bacteroides |  | U>R |  | U>R | U>R |  | U>R |
| 203 | f_Ruminococcaceae |  |  |  |  |  |  | R>U |
| 204 | g_Bifidobacterium |  |  |  | U>R |  |  |  |
| 205 | g_Bacteroides |  | U>R |  | U>R | U>R |  | U>R |
| 206 | s_Bacteroides plebeius |  |  |  | U>R | U>R |  | U>R |
| 207 | g_Pseudoramibacter_Eubacterium |  | R>U |  | R>U | R>U |  | R>U |
| 212 | f_Lachnospiraceae |  |  |  |  |  | R>U |  |
| 213 | g_Phascolarctobacterium |  |  |  |  |  |  | R>U |
| 216 | s_Bacteroides uniformis |  |  |  | U>R |  |  |  |
| 217 | s_[Ruminococcus] gnavus | R>U |  |  |  |  | R>U |  |
| 218 | s_[Ruminococcus] gnavus |  |  |  |  | U>R |  |  |
| 220 | o_RF32 |  | R>U |  | R>U | R>U |  | R>U |
| 234 | g_Corynebacterium |  | U>R |  | U>R |  |  |  |
| 239 | f_Caulobacteraceae |  |  | R>U |  |  |  |  |
| 245 | o_RF32 |  | R>U |  | R>U | R>U |  |  |
| 247 | f_[Barnesiellaceae] |  |  |  | R>U | R>U |  |  |
| 261 | f_S24U>R7 |  | R>U |  |  |  |  |  |
| 274 | g_Morganella |  | U>R | U>R |  |  |  |  |

*, R>U, More predominant in rural than in urban samples. U>R, More predominant in urban than in rural samples. IM: ileal mucosa; ID: ileal digesta; CM: colon mucosa; CD: colon digesta; FS1: feces collected after first inoculation; FSN: feces collected during necropsy.
